# Decomposing working memory subprocesses with the reference-back paradigm: Event-related potentials and age-related differences

**DOI:** 10.1371/journal.pone.0307351

**Published:** 2024-12-04

**Authors:** Zsófia Anna Gaál, Boglárka Nagy, István Czigler, Petra Csizmadia, Béla Petró, Petia Kojouharova

**Affiliations:** Institute of Cognitive Neuroscience and Psychology, HUN-REN Research Centre for Natural Sciences, Budapest, Hungary; Education University of Hong Kong, HONG KONG

## Abstract

We used a data-driven approach to study the electrophysiological correlates of the working memory subprocesses revealed by the reference-back paradigm. In the absence of prior research, we focused on how aging affects the four subprocesses: *updating*, *substitution*, *gate opening*, and *gate closing*. We conducted our experiment with 24 younger adults (M = 20.17±1.47) and 23 older adults (M = 67.35±4.01). Significant reaction time costs were observed for all four subprocesses, but age-related differences were found only in *substitution*, which was larger in older than younger adults, indicating it as being the most vulnerable subprocess in aging. Using difference waves, we identified event-related potential components that characterize the subprocesses we studied. Regarding *updating*: three occipital negativities between 80–180 ms, 300–400 ms, and 400–1,000 ms were observed, with only the latter range showing age group differences. Source analysis showed larger activity differences in the right frontal and temporal areas for younger adults. Regarding *substitution*: a frontal positivity between 250–600 ms emerged in younger adults, while a posterior positivity between 550–750 ms was found in older adults indicating different underlying processes supported by sLORETA results. Regarding *gate opening*: three parieto-occipital components were identified: a negativity between 150–250 ms, a positivity between 300–500 ms, and a positivity between 500–700 ms, all showing age-related differences. Regarding *gate closing*: we found an occipital negativity between 150–300 ms and a frontal positivity between 300–600 ms, neither of which changed between the age groups. From our findings, we conclude that the process of protecting information (*gate closing*) remains stable with age, despite older adults’ sensitivity to interference. Conversely, we observed age-related differences in *gate opening*, which may have led to different strategies being used by the two age groups during *substitution* (the process by which new information is incorporated into working memory), as reflected in the distinct brain activity patterns observed.

## 1. Introduction

Working memory plays a crucial role in maintaining and manipulating short-term information and contributing to other cognitive processes such as learning, decision-making, and problem-solving. For this reason, numerous studies aim to better understand its mechanisms. This knowledge is crucial when, for any reason, the functioning of the nervous system changes, as it helps us identify the causes of these changes and find ways to compensate for them. An example of such a situation, as presented in our study, is aging, where one of the most well-known and concerning changes is the decline in working memory [1]. To better understand the reasons behind the changes in working memory, a possible approach is to attempt to separate its individual subprocesses and examine them independently, along with the underlying brain structures. This allows us to investigate whether one or more subprocesses are affected and which ones play a role in the deteriorated function of working memory.

**Fig 1:**
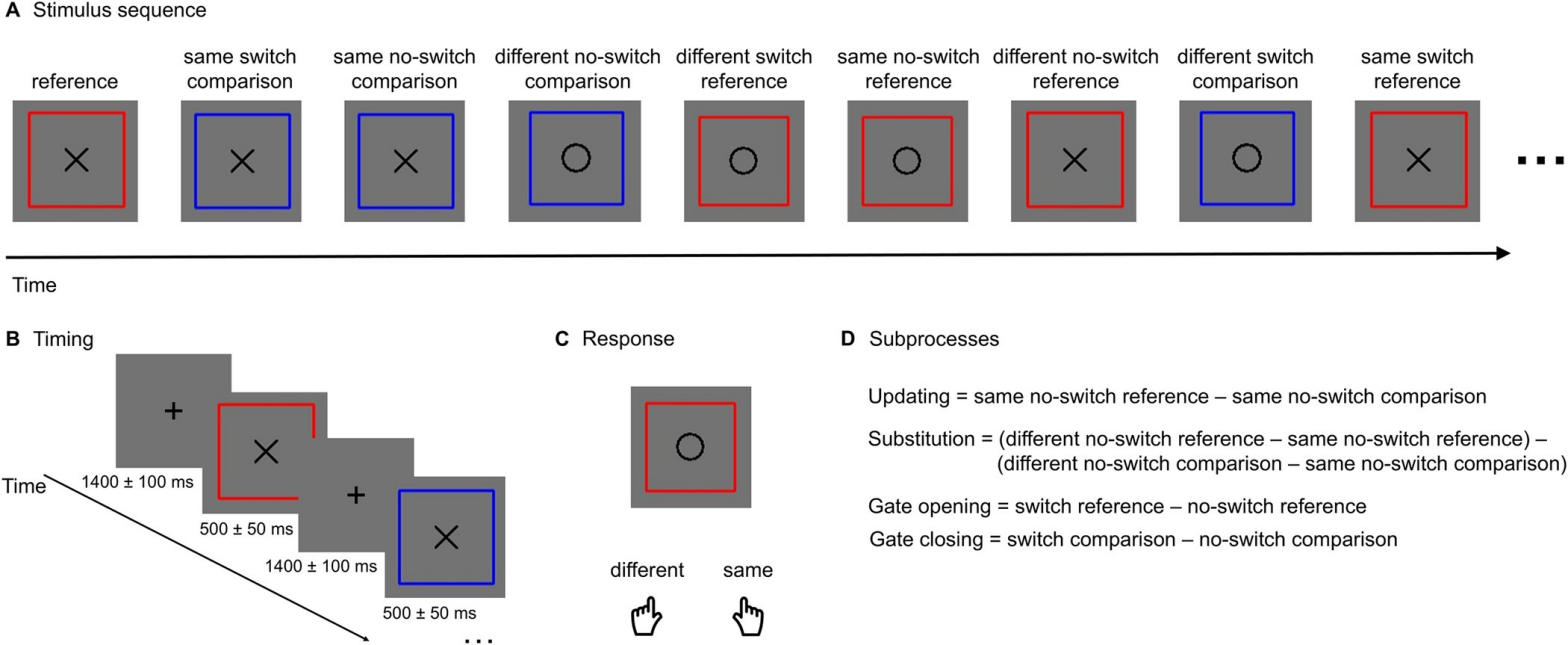
**A** A stimulus sequence example consisting of reference trials (red frame) and comparison trials (blue frame). The target stimulus was the letter X or the letter O displayed in the centre of the frame. **B** The stimulus duration was 500 ms (±50 ms). The inter-stimulus interval (ISI) duration was 1400 ms (±100 ms) during which a fixation cross was visible in the centre of the screen. **C** A response was given by pressing the left key if the target letter in the trial was different from the reference, and the right key if the target letter was the same.

A potential method for this exploration is provided by the reference-back paradigm developed by Rac-Lubashevsky and Kessler [2]. In this working memory model, the central element is a gating mechanism which regulates the protection and updating of content in the working memory. The reference-back paradigm allows for the separation of the subprocesses of working memory (which we indicated in italics throughout the manuscript)–*updating*, *substitution*, *gate opening*, and *gate closing*–by manipulating the state of the gate and the need to update in the individual trials. This is a modified version of the n-back paradigm: letters (X and O) are presented randomly in succession within either a red or a blue frame, and the task is to determine whether the currently presented letter is identical or not to the last letter presented within a red frame. So, trials with a blue frame (comparison trials) require only a matching decision (the gate is closed); whilst trials with a red frame (reference trials) also require the updating of the content in working memory (the gate is open). As an example (Fig 1): if an X appears in a red frame in the first trial, it will serve as a reference until the next red frame trial, and the participant will compare the subsequent letter to this one. In the second trial, an X is shown in a blue frame, indicating a comparison trial: the gate closes to protect the content of the WM, as only a matching decision is required without updating. In next trial, an O is presented in a blue frame, prompting another matching decision–whether the O is similar to the last letter in the red frame–and the gate remains closed. However, in the fourth trial, the O appears in a red frame: it will be compared to the last red-framed letter (X), as in the previous two trials, but this time not only is a matching decision made, but the content of the working memory are also updated, enabled by gate opening, making this letter the new reference until the next red frame. While in this trial an update with new information occurs–a *substitution* (O replaces X)–, in the next trial, where an O appears in a red frame, only *updating* takes place, still incurring a cost even when there is no mismatch between two consecutive reference trials.

Behavioural results from studies utilizing the reference-back paradigm [2, 3] indicate the following: 1) an *updating cost* with reaction times being slower for reference compared to comparison trials; 2) a *substitution cost* where reaction times are much slower for “different” compared to “same” responses in reference trials to comparison ones; 3) a *gate opening cost* where reaction times are slower for reference trials following a comparison trial (switch) than reference trials following another reference trial (no-switch); and 4) a *gate closing cost* where reaction times are slower for comparison trials following a reference trial (switch) than comparison trials following another comparison trial (no-switch). These results confirmed the separability and examinability of the presumed subprocesses [2, 3].

The above subprocesses, according to the authors, can be matched with the biological processes hypothesized by the prefrontal cortex basal ganglia working memory model (PBWM [4–6]). The basal ganglia serve the gating function, separating sensory input in the sensory cortex; or internally stored input in long-term memory from the working memory representation maintained in the dorsolateral prefrontal cortex. In the baseline state, the gate is closed, preserving the content of working memory. However, when required by the task, the gate opens, allowing for the working memory to update with new information. By this the inhibitory input from the basal ganglia to the thalamus, which has bidirectional excitatory connections with the frontal cortex, is released. This mechanism enables control over working memory content: flexibly alternating between either protecting or refreshing it with new information.

The assumed biological basis of the model, the involvement of the structures mentioned above in working memory processes, is supported by fMRI results. Nir-Cohen and her colleagues [3] employed a reference-back task using faces instead of letters. Their results corroborated the involvement of the basal ganglia-thalamus-prefrontal cortex circuits with some refinements. The basal ganglia (caudate, putamen and pallidum) exhibited increased activity for *gate opening* and *substitution*, but not for *gate closing* and *updating*. Enhanced thalamic activity was identified solely for *gate opening*. The entire frontoparietal network, including the dorsolateral prefrontal cortex, the medial prefrontal cortex, and the posterior parietal cortex, was activated during *gate opening*; while a lateralized activity in the left parietal and frontal cortex was observed during *substitution*. *Updating* was associated with activity increase in the posterior parietal cortex. Pronounced activity was also noted in the fusiform face area during *gate opening* and *substitution*. In summary, the four subprocesses were linked to distinct neural activity patterns. While *gate opening* showed an activation pattern supporting the PBWM model, these structures were not involved in *gate closing*. Additionally, other structures, such as the posterior parietal cortex and fusiform face area, were also involved in working memory *updating*. The posterior parietal cortex is known to be crucial for various aspects of working memory [7–9], while the fusiform face area is a critical region for face processing [10] (note that in this study the stimuli were not letters but faces). However, a reanalysis of these results [11] revealed frontal, striatal, and thalamic activity during *gate closing* when the current stimulus differed from the one held in working memory, and no activity increase was observed except when the current stimulus and the one held in working memory were identical. Thus, active gate closure occurred only when it was necessary to shield the information, and not by default. Also, selective gating was observed for *gate opening*, occurring only when genuinely necessary and not during task repetition. Another study has suggested that similar neural patterns are activated regardless of whether working memory is updated with declarative or procedural content [11].

While fMRI allows us to explore which brain regions are involved in individual subprocesses, the event-related potential (ERP) method enables us to examine their temporal dynamics with millisecond precision. It is important to examine ERP data in addition to behavioural data because the two levels do not necessarily reveal the same information; they can complement each other or highlight differences that may not be observable at one level. This is especially relevant when comparing two different groups. For example, we have findings where the same effect was observed at the behavioural level in both young and older adults, while ERP revealed that they achieved similar results through different mechanisms [12]. To date, only two studies [13, 14] have investigated the reference-back task with ERPs. Rac-Lubashevsky and Kessler [13] focused solely on the P3b component. The reasoning for this is that the P3b component is associated with updating the content of working memory [15]; its amplitude increases when a stimulus is relevant to the current task, yet unexpected. However, Rac-Lubashevsky and Kessler [13] concluded that the P3b was not associated with working memory updating; but rather indicated target categorization. Although Csizmadia and her colleagues [14] identified several components that played a role, they focused only on gate opening, and specifically on the differences between groups with different levels of divergent and convergent thinking.

Nevertheless, it is of note the authors in these studies drew their conclusions from the original ERPs, even though their primary concept for distinguishing working memory subprocesses relied on subtracting specific trial types. When working with ERPs, utilizing difference waves is proven to be more effective in isolating processes that exhibit distinct activity between two (or more) trial types. This approach helps to eliminate patterns that show no variation between these trial types, while emphasizing the relevant components that result from their distinct activation patterns. Consequently, this method enables us to make specific conclusions based on the understanding of how these subprocesses are uniquely activated in different trial types [16]. Therefore, in the current study, we employed difference waves, and we used a data-driven approach (cluster-based permutation t-tests). The purpose of the cluster-based permutation analysis was twofold: 1) to establish the presence of the subprocess (i.e., the difference between the conditions) in the difference potentials, and 2) to inform further analysis by estimating the possible time windows and locations where components unique to each subprocess could be observed. In this regard, our study was exploratory in nature.

In addition to exploring which subprocesses manifest in each component, we aimed to understand how these subprocesses differ between young and older adults. In the reference-back task, as mentioned above, only the P300 component has been investigated so far. This component typically appears with longer latency, smaller amplitude, and a different distribution in older adults compared to younger ones: while younger individuals display a parietal maximum, older adults show a more even distribution and may also exhibit a frontal peak [17–19]. In the n-back task–which is structurally very similar to the reference-back task: the participant needs to decide whether the current stimulus matches the one presented n trials earlier–studies have also focused on the P300 component. The results indicate that the component’s amplitude decreases with increased WM load [20–22] and shows an age-related decline as well [21, 22]. Lubitz and colleagues [23] also investigated the P200 component in this task. They found that the P200, which they interpret as a marker of executive attention, had a higher amplitude in the 2-back task compared to the 1-back task in younger adults. In low-performing older adults, the opposite effect was observed, while in high-performing older adults, there was no difference in amplitude between the two tasks. For the P300 component, the amplitude decreased with increased WM load in all groups and was higher parietally in younger adults than in older adults. Additionally, in high-performing older adults, a frontal increase was found, which was interpreted as a compensatory process. Based on the above, we can assume that the P300 component primarily appears in the updating and substitution subprocesses, which are expected to show smaller amplitudes and more uniform distribution in older adults.

There are several theories about the neural processes that may underlie the age-related decline in working memory (WM). Some theories attribute the decline to more general processes, such as overall slowing [24] or reduced inhibitory processes [25]. The latter suggests that reduced inhibitory function leads to irrelevant elements being included in WM, causing interference and reducing performance [26]. Other theories explain the decline by the altered functioning of specific brain regions, particularly highlighting the role of the prefrontal cortex (PFC). A key theory is the HAROLD model [26], which posits that PFC activity in older adults is less lateralized compared to younger adults, potentially due to a dedifferentiation process or compensatory activation of the contralateral area to offset weaker functioning. The CRUNCH (Compensation-Related Utilization of Neural Circuits Hypothesis, [27]) and STAC (Scaffolding Theory of Aging and Cognition, [28]) models also emphasize compensation in response to declining neural structure and function. Overall, changes in frontal cortex functioning with age have been described in numerous studies [e.g., 29–32].

Taking into consideration that the macrostructure, microstructure, and neural connectivity of each involved structure change with aging (basal ganglia [33]; thalamus [34, 35]; prefrontal cortex [36, 37]); it is expected that individual subprocesses will also exhibit differences between young and older adults, primarily in *gate opening* and *substitution*. This is because these subprocesses show the most significant activity increase in these structures. We also applied source localisation analysis to identify possible differences in the sources of the subprocesses between younger and older adults. Any differences in the ERPs can reflect differences in the use of brain resources, and source localisation can help to identify the brain structures responsible for these differences. Furthermore, based on the results that older adults have difficulty excluding irrelevant elements from working memory, we can hypothesize that the gate closing subprocess does not function with optimal efficiency.

To summarize, our study investigated the subprocesses involved in working memory and also their temporal course in the reference-back paradigm. Our aim was to identify the components of each subprocess using a data-driven approach, as earlier studies have neither done this; nor have utilized difference waves measurement. Furthermore, we endeavoured to examine how these subprocesses may change with aging.

## 2. Material and methods

### 2.1. Participants

Two age-groups participated in the experiment. There were 29 participants in each group. Five participants in the older group did not complete the session, and a further one was excluded because of a lack of valid epochs in one of the conditions. Two participants in the younger group were excluded because of recording issues, and three others were excluded because of too few epochs in some of the conditions. In the final sample, the younger group consisted of 24 participants (mean age: 20.17, SD = 1.47, range 18–23 years; 16 women; 2 left-handed), and the older group consisted of 23 participants (mean age: 67.35, SD = 4.01, range: 60–74 years; 14 women; 1 ambidextrous). To exclude participants whose cognitive functioning may indicate impairment (such as dementia), we measured the four major components of intelligence with four subtests of the Hungarian version of Wechsler Adult Intelligence Scale (WAIS-IV [38]) representing the four major components: Similarities–verbal comprehension; Digit Span–working memory; Matrix Reasoning–perceptual reasoning; and Coding–processing speed. The scaled scores (where the age-group average is 10) achieved by the younger group were as follows: Similarities: M = 11 (SD = 2.3); Digit span: M = 8.7 (SD = 2.4); Matrix Reasoning: M = 10.3 (SD = 1.8); and Coding: M = 11.3 (SD = 2.8). The older group achieved the following scores on the four subtests: Similarities: M = 13.5 (SD = 2.0); Digit Span: M = 11.4 (SD = 3.4); matrix reasoning: M = 12.8 (SD = 2.9); and Coding: M = 14.2 (SD = 3.0). Every participant had normal or corrected-to-normal vision (at least 5/5 in a version of the Snellen charts), and self-reported no history of any neurological or psychiatric disorder. All participants were paid for their contribution and were recruited between October 2018 and March 2019. The protocol was approved by the Joint Psychological Research Ethics Committee (EPKEB, Hungary) and a written informed consent was obtained from all participants.

### 2.2. Stimuli and procedure

Each participant was seated in a comfortable chair in a sound-attenuated and electrically shielded chamber. Stimuli presentation and behavioural data collection were conducted with MATLAB R2014a [39]. The stimuli were presented on a 21.5-inch LCD monitor (Asus VS229na, 60 Hz refresh rate) at a 1.4 m distance. The present study was composed of one session in which the tasks were always presented in the same order: a forced-choice task; a reference-back task; a reference-back task with distractors; and a memory task where participants had to indicate if they remembered the distractor stimuli. The present paper considers only the reference-back task, and its experimental design is shown in Fig 1. The stimuli (letter “X” at 0.35° visual angle; or “O” at 0.4° visual angle) were presented in 4 pixel-wide lines against a grey background (RGB (0.45,0.45,0.45)) within either a red or a blue frame at 1.15˚ visual angle. Each trial started with a fixation-cross that was presented for 1,400 ms (with +/-100 ms jitter in 16.6 ms steps), followed by one of the stimuli for 500 ms (with +/-50 ms jitter in 16.6 ms steps). Participants had the time from the presentation of one stimulus until the appearance of the next to respond, which corresponded to a 1,900±150 ms time window. The trials were presented in a randomized order in blocks, with balanced proportions of the trial types across blocks that varied within an average deviation of 1–1.5 trials. Each block contained 80 trials, and nine blocks were presented for a total of 720 trials. The experimental blocks were preceded by 4 practice blocks (15 trials per block). Throughout the experimental blocks, participants were asked to keep their gaze on the centre of the screen. The task had two trial types: in reference trials (probability 0.5) the letters appeared within a red frame, and in comparison trials (probability 0.5) they were presented within a blue frame (see: Fig 1). A reference trial could follow a comparison trial (switch reference, the gate opens, switch trial) or another reference trial (no-switch reference, the gate remains open, no-switch trial). Similarly, a comparison trial could follow a reference trial (switch comparison, the gate closes, switch trial) or another comparison trial (no-switch comparison, the gate remains closed, no-switch trial). Within a block the probability of a no-switch trial was 0.75, and the probability of a switch trial was 0.25. In each trial, participants had to select whether the stimulus was or was not the same as the one in the last seen reference trial (red frame). “Same” responses, on a modified computer keyboard with only two keys, were indicated by pressing the ‘L’ key with the right index finger; and “different” responses, by pressing the ‘A’ key with the left index finger. Within all blocks of trials an equal number of “same” and “different” responses were required. After each block, participants received feedback on their performance, including: the average reaction time; the number of correct responses; and the number of errors. The feedback helped participants track their performance and served as an incentive to respond as quickly and accurately as possible. Responses below 150 ms were considered invalid as such short response latency indicates that the response begun before the presentation of the stimulus, and were thus excluded from data analysis (a total of 62 trials for the experiment).

### 2.3. ERP recording

EEG was recorded with BrainVision actiCHamp amplifier (Brain Products GMBH, DC-280 Hz, sampling rate: 1,000 Hz). Brain activity was recorded from 32 Ag/AgCl active electrodes, in accordance with the extended 10–20 system (F7, F3, Fz, F4, F8, FC3, FC4, T7, C3, Cz, C4, T8, CP5, CP6, P7, P3, Pz, P4, P8, PO7, PO3, POz, PO4, PO8, O1, Oz, O2, with AFz as the ground) using an elastic electrode cap (EasyCap, Brain Products GMBH). The reference electrode was placed on the tip of the nose. Horizontal EOG was recorded with a bipolar configuration between electrodes positioned lateral to the outer canthi of the eyes (one electrode on each side). Vertical eye movement was monitored with a bipolar montage between two electrodes, one placed above and one below the left eye. The impedance of the electrodes was kept below 10 kΩ.

### 2.4. Data analysis

#### 2.4.1. Computation of the subprocesses

The subprocesses were computed as follows: 1) *updating* = no-switch reference–minus–no-switch comparison, where information is entered into working memory independent of whether it is the same as the maintained information or not; 2) *substitution* = (different no-switch reference–minus–same no-switch reference)–minus–(different no-switch comparison–minus–same no-switch comparison), where the information in working memory needs to be replaced only if the reference changes; 3) *gate opening* = switch reference–minus–no-switch reference, where the gate to working memory needs to be opened opposite to being maintained open; 4) *gate closing* = switch comparison–minus–no-switch comparison, where the gate needs to be closed opposite to being maintained closed. The computations followed Nir-Cohen and colleagues’ [3] paper where switch trials were excluded from the *updating* and *substitution* calculations so that additional processes such *gate opening* and *gate closing* would not bias the results. These computations are also illustrated in Table 1. In our analysis, these computations are applied for comparing the size of the behavioural effects between the age groups as well as for calculating the difference ERP potentials.

**Table 1:** Calculation of the subprocesses in the reference-back task.

TRIAL TYPE	Reference				Comparison			
GATE SWITCH	No-switch		Switch		No-switch		Switch	
RESPONSE	Same	Different	Same	Different	Same	Different	Same	Different
Gate opening	-	-	+	+				
Gate closing					-	-	+	+
Substitution	-	+			+	-		
Updating	+	+			-	-		

#### 2.4.2. Behavioural data analysis

First, we calculated overall mean performance (percentage of correct responses of all trials) and overall mean reaction time (for correct responses only) to assess whether there was an overall difference between the age groups in the task. Because of the response time limit, outlier RTs were not removed (under 1% of RTs were between 1,500 ms and 2,000 ms in either age group). While slower reaction time (RT) was expected in the older group, we wanted to ensure that the level of performance was similar in the groups. Furthermore, in a task with a time limit there are two possible types of error: either an incorrect response is given within the allowed time limit, or there is no response (miss). So, overall incorrect response rate and misses rate were also analysed.

Mixed analyses of variance (ANOVAs) on the mean RT results for the correct responses were performed to explore the presence of the investigated effects as well as possible differences between the age groups, following the approach used in previous studies [2, 3, 13]. Only RTs for correct responses were included in the averages (see Table 1 for the factors and levels included in each ANOVA). For the *updating* subprocess, we conducted an ANOVA with TRIAL TYPE (no-switch reference, no-switch comparison) as the within-subject factor and AGE GROUP (younger, older) as the between-subject factor. A statistically significant main effect of TRIAL TYPE means presence of the *updating cost*, while a significant TRIAL TYPE × AGE GROUP interaction means a difference in the *updating cost* between the age groups. For the *substitution* subprocess, we conducted an ANOVA with TRIAL TYPE (no-switch reference, no-switch comparison) and RESPONSE (same, different) as the within-subject factors and AGE GROUP (younger, older) as the between-subject factor. A significant TRIAL TYPE × RESPONSE interaction suggests the presence of *substitution*, while a significant TRIAL TYPE × RESPONSE × AGE GROUP interaction suggests a difference in the subprocess between the age groups. For the *gate opening* subprocess, we conducted an ANOVA with GATE SWITCH (switch reference, no-switch reference) as the within-subject factor and AGE GROUP (younger, older) as the between-subject factor. A significant main effect of GATE SWITCH may indicate the presence of *gate opening*, while a significant GATE SWITCH × AGE GROUP interaction supports an assumption for an age-based difference in the subprocess. Lastly, for the *gate closing* subprocess, we conducted an ANOVA with GATE SWITCH (switch comparison, no-switch comparison) as the within-subject factor and AGE GROUP (younger, older) as the between-subject factor. A significant main effect of GATE SWITCH means a presence of *gate closing*, while a significant GATE SWITCH × AGE GROUP interaction suggests a difference in the subprocess between the age groups.

While the ANOVA analyses can show the presence of a subprocess and a difference between the age groups, and the *post hoc* tests can indicate the differences between the different conditions, a more direct comparison of the size of the effects would be to compare the difference in mean RT between the conditions. The RT differences were computed according to Table 1 and then compared between the age groups.

A case could be made that instead of the simple RT differences, relative RT differences should be analysed between the groups as the older group is relatively slower overall compared to the younger group. This argument holds if the investigated process also slows down with aging, which is a possibility. Therefore, we also calculated a RT ratio index for each participant, which was the RT difference between the conditions divided by the average RT for the conditions (the sum divided by two). In the case of *substitution*, two ratios were calculated (reference and comparison), and then subtracted.

Similar analyses were performed separately for incorrect responses and misses. The results aligned with the RT results and a summary can be found in the S1 File. A full report of the RT results can be found in S2 File.

#### 2.4.3. ERP preprocessing and data analysis

The EEG data was filtered offline with a non-causal Kaiser-windowed Finite Impulse Response filter (low pass filter parameters: 30 Hz cut off frequency, beta of 12.2653, a transition bandwidth of 10 Hz; high pass filter parameters: 0.1 Hz cut off frequency, beta of 5.6533, a transition bandwidth of 0.2 Hz). Independent Component Analysis (ICA) was applied on the filtered EEG data in order to reject eye-movement artifacts (blinking, horizontal eye movements). The EEG was then segmented into epochs of 1,100 ms from 100 ms pre-stimulus to 1,000 ms post-stimulus. The mean voltage during the 100 ms pre-stimulus interval served as the baseline for amplitude measurements. Stimulus onset was measured by a photodiode, providing exact zero value for averaging. Epochs were rejected if they had a larger than 100 μV voltage change between the minimum and maximum of the epoch on any channel.

Epochs for trials with correct responses were averaged per participant for each condition relevant to the calculation of the effects, and then difference potentials were computed according to Table 1: 1) *updating* = no-switch reference ERP–minus–no-switch comparison ERP, 2) *substitution* = (different no-switch reference ERP–minus–same no-switch reference ERP)–minus–(different no-switch comparison ERP–minus–same no-switch comparison ERP), 3) *gate opening* = switch reference ERP–minus–no-switch reference ERP, 4) *gate closing* = switch comparison ERP–minus–no-switch comparison ERP. Difference potentials were used for the following reasons: 1) latent components that could be present across more than one ERP component are better identified [16], 2) reduced number of factors in the ANOVA analysis which reduces the possibility for meaningless interactions and chance results [40], 3) better suited for an exploratory cluster-based permutation analysis (see below). The average number of epochs per condition for each participant can be found in Table 2.

**Table 2:** Mean number and ranges of the epochs per condition per participant in each age group. Only correct trials were included in the average. Standard deviation is indicated in parentheses. The ranges (min-max) are shown in the second row of each cell.

Conditions			AGE GROUP	
TRIAL TYPE	GATE SWITCH	RESPONSE	Younger group	Older group
Reference	No-switch	Same	104.08 (16.62)	113.17 (10.49)
			63–129	91–129
		Different	93.36 (21.00)	85.96 (17.35)
			35–112	47–112
	Switch	Same	40.71 (7.48)	43 (5.56)
			25–51	28–51
		Different	31.79 (6.29)	25.26 (7.00)
			19–47	11–47
Comparison	No-switch	Same	100.17 (19.82)	100.57 (12.93)
			51–128	66–128
		Different	103.92 (20.28)	107.61 (14.36)
			50–131	59–131
	Switch	Same	38.71 (5.87)	39 (4.57)
			26–45	27–45
		Different	40.29 (8.05)	40.52 (5.22)
			21–51	30–51

First, a cluster-based permutation *t*-test analysis was conducted on each difference potential within the groups for electrode sites F3, Fz, F4, C3, Cz, C4, P3, Pz, P4, O1, Oz, O2 and for all time points after stimulus onset (0–1,000 ms). The threshold *p*-value was set at .05, and the familywise error (FWER) correction was set at .05. We used *t*_*max*_ statistics. For each analysis, 10,000 permutations were run, and the tests were two-tailed to identify both positive and negative clusters. The cluster-based permutation analysis was chosen as an exploratory approach as to when (e.g., early or late processes) and where (e.g., anteriorly or posteriorly) the investigated effect was present (if at all) in each age group. Here, it is important to note that cluster-based permutation analysis is often overinterpreted [41, 42]. This analysis shows that there is a difference between the compared conditions in the chosen time window, but any report of the location and the extend of the clusters is purely descriptive, imprecise, and may only be used to inform later decisions about the analysis. In this study, the whole epoch excluding the baseline as well as electrode sites along the midline were included to observe where clusters may be formed. However, the “where” and “when” are mostly basis for further analysis. The full results are presented in the S3 File.

Next, we identified time windows in which to compare the difference potentials between the age groups. The time windows were selected based on the results from the cluster-based permutation analysis and visual inspection of the difference potentials (see Results). In the visual inspection two criteria were considered: 1) components were identifiable in the difference potentials, and 2) the 95% confidence intervals around the difference potentials indicated significant differences from zero (i.e., the confidence interval did not cross the x axis) for multiple consecutive data points within the time window. Possible components that begun after the time window for the average RT were considered unrelated to WM processes and thus were outside the scope of this study. For *updating*, the selected time windows were 80–180 ms, 300–400 ms, and 400–1,000 ms post-stimulus. For *substitution*, the selected time windows were 250–600 ms and 550–750 ms post-stimulus. For *gate opening*, the selected time windows were 150–250 ms, 300–500 ms, and 500–700 ms post-stimulus. For *gate closing*, the selected time windows were 150–300 ms and 300–600 ms post-stimulus. Mixed ANOVAs were performed for each condition and each time window across the midline with ANTERIORITY (Fz, Cz, Pz, Oz) as the within-subject factor and AGE GROUP (younger, older) as the between-subject factor. The inclusion of ANTERIORITY as an additional factor was based on the assumption that components may have different scalp distribution in the two age groups. A full report of the results can be found in S4 File.

EEG data were preprocessed with MATLAB R2015a [43] and EEGLAB [44]. ICA was performed with the runica function of the ERPLAB toolbox [45]. A cluster-based permutation *t*-test analysis was conducted on each difference potential within the groups with the Mass Univariate ERP Toolbox [46].

#### 2.4.4. sLORETA analysis

We applied a distributed source localisation technique to locate and compare the cortical sources for potential differences between the groups. The source signal of the average ERP time series was reconstructed on the cortical surface by applying the sLORETA inverse solution [47]. The sLORETA gives a solution for the EEG inverse problem by applying a weighted minimum norm estimation with spatial smoothing and standardization of the current density map. The forward model was generated on a realistic BEM head model [48] by applying a template MRI (ICBM152; 1 mm3 voxel resolution) with template electrode positions. The reconstructed dipoles (pA/m) were determined for every 15,002 sources in three orthogonal directions (unconstrained solution). For each subject and each subprocess, the sources for the respective ERPs (see ERP preprocessing and data analysis) were estimated, their difference computed, then normalized to baseline and flattened.

For the comparison between the groups non-parametric two-sample independent t-tests were conducted on the source differences for the same time intervals as defined in the difference potential analysis, and FDR correction (alpha = .01) was applied [49]. Only differences in at least five voxels were considered. Brain regions for the corresponding significant activations were identified based on the parcellation scheme introduced by Klein and Tourville [50]. A source localisation analysis regarding the sources of the effects within each group can be found in the S5 File.

The sLORETA analysis was performed with Brainstorm [51]. Group analysis was conducted according to the Group analysis processing pipeline described in Tadel et al. [49].

#### 2.4.5. Bayesian analysis

There was no *a priori* power analysis to calculate the size of the sample, thus Bayesian analyses were also conducted to evaluate the strength of the evidence for either the null or the alternative hypothesis based on the current sample [52, 53]. This was the main goal for including this analysis. The default prior distributions for ANOVA in JASP were used. Specifically, fixed effects had an *r* scale of 0.5, and random effects had an *r* scale of 1. Factors were compared across matched models. We also used the default prior option for the *t*-tests and the Mann-Whitney nonparametric tests, a Cauchy distribution with spread *r* set to 0.707. All tests were two-tailed.

A Bayesian Factor (BF) larger than 3 indicates evidence for the alternative hypothesis or for the inclusion of a main effect or an interaction (predictor) in the model, while a BF smaller than 0.333 suggests evidence for the null hypothesis or the exclusion of the main effect or the interaction from the model. When an effect or a difference are stated to be statistically significant and no further comment is made within the text, then the evidence is at least moderate in favour of the alternative hypothesis or the inclusion in the model. BF_10_ is reported for the paired tests, i.e., when the likelihood of the data under the alternative hypothesis is compared to the likelihood of the data under the null hypothesis. BF_incl_ is reported for the main effects and the interactions to indicate the impact of each predictor in the model. Regarding the ANOVA results, the value of interest is BF_incl_ as it provides evidence for the contribution of a factor to the model’s variance.

The statistical analyses including the Bayesian analysis were performed with JASP [54]. The Greenhouse-Geisser corrections were used when necessary. For *post-hoc* comparisons the Bonferroni correction was applied. For the comparisons of the size of the effects between the groups an independent *t*-test or a Mann-Whitney U nonparametric test was applied depending on the assumption of normality.

## 3. Results

The mean RTs for each condition and age group can be found in Table 3. The mean amplitudes for each difference potential, time window, and age group are displayed in Table 4. The relevant statistical behavioural and ERP results are summarised in Tables 5 and 6, respectively. The results from the source localisation analyses are summarised in Table 7. Full details regarding the descriptive statistics and the statistical results can be found in the S1, S2, and S4 Files.

**Table 3:** Mean reaction time for correct trials per condition in each age group. Standard deviation is indicated in parentheses.

Conditions			AGE GROUP	
TRIAL TYPE	GATE SWITCH	RESPONSE	Younger group	Older group
Overall			521.76 (97.01)	652.41 (118.5)
Reference	No-switch	Same	476.76 (91.22)	577.61 (113.75)
		Different	591.57 (109.49)	775.32 (148.79)
	Switch	Same	508.87 (125.89)	616.38 (128.51)
		Different	625.23 (147.91)	846.95 (170.7)
Comparison	No-switch	Same	451.32 (82.91)	578.25 (106.35)
		Different	510.72 (83.95)	652.72 (114.83)
	Switch	Same	526.05 (120.78)	613.73 (122.36)
		Different	620 (157.48)	742.3 (140.36)

**Table 4:** Mean amplitude per effect for each time window and each electrode site in each age group. Standard deviation is indicated in parentheses.

	Age group	Younger group				Older group			
	Electrode site	Fz	Cz	Pz	Oz	Fz	Cz	Pz	Oz
Effect	Time window								
Updating	80–180 ms	0.37 (0.73)	-0.42 (0.74)	-0.88 (1.79)	-1.91 (2.22)	0.17 (0.47)	-0.198 (0.44)	-0.44 (0.87)	-0.996 (1.47)
	300–400 ms	0.01 (0.4)	-0.15 (0.49)	-0.15 (0.9)	-1.995 (1.39)	0.12 (0.32)	-0.07 (0.29)	-0.08 (0.56)	-0.75 (0.85)
	400–1,000 ms	0.08 (0.29)	-0.14 (0.26)	-0.62 (0.55)	-1.795 (1.15)	-0.12 (0.24)	-0.02 (0.21)	-0.26 (0.51)	-1.67 (0.84)
Substitution	250–600 ms	0.79 (0.84)	-0.28 (0.96)	-0.40 (1.69)	0.45 (2.02)	0.06 (0.72)	0.13 (0.54)	0.48 (1.45)	0.48 (1.72)
	550–750 ms	0.36 (0.95)	-0.07 (1.10)	-0.03 (2.15)	-0.08 (2.67)	0.42 (0.62)	-0.06 (0.80)	1.01 (1.81)	1.58 (2.07)
Gate opening	150–250 ms	-0.3 (0.48)	0.27 (0.74)	0.43 (1.48)	-0.21 (1.7)	-0.06 (0.69)	-0.28 (0.51)	-0.68 (1.14)	-1.66 (1.41)
	300–500 ms	-0.44 (0.66)	0.76 (0.9)	1.86 (2.27)	1.45 (2.82)	0.01 (0.56)	0.04 (0.52)	0.32 (1.17)	-0.08 (1.53)
	500–700 ms	-0.33 (0.68)	0.64 (0.78)	1.4 (1.88)	1.46 (2.65)	0.2 (0.52)	0.06 (0.5)	0.56 (1.08)	0.75 (1.16)
Gate closing	150–300 ms	0.7 (0.56)	-0.16 (0.55)	-0.42 (0.93)	-0.92 (1.28)	-0.09 (0.34)	-0.12 (0.44)	-0.37 (0.99)	-0.91 (1.15)
	300–600 ms	0.42 (0.62)	-0.03 (0.57)	0.26 (1.57)	-0.36 (1.8)	0.32 (0.52)	-0.34 (0.45)	-0.12 (0.96)	0.05 (1.44)

**Table 5:** A summary of the behavioural statistical results regarding the overall presence of the subprocess, the differences in the subprocess between the groups (repeated measures ANOVA results), and the differences in the size of the effects (independent groups comparisons). The ms column indicates the RT difference.

**Overall presence of the effect**												
Condition		Calculation	ms		*F*(1,45)		*P*		η_p_^2^		BF_incl_	
Updating		Main effect of TRIAL TYPE	46.54		43.142		< .001		.489		195259.14	
Substitution		TRIAL TYPE × RESPONSE interaction	88.6		90.103		< .001		.667		8.159×10^10^	
Gate opening		Main effect of GATE SWITCH (reference)	36.32		23.215		< .001		.34		827.905	
Gate closing		Main effect of GATE SWITCH (comparison)	77.66		72.952		< .001		.618		5.316×10^7^	
**Overall difference between the groups**												
Condition		Calculation	ms		*F*(1,45)		*P*		η_p_^2^		BF_incl_	
Updating		Main effect of AGE	133.12		19.468		< .001		.302		179.056	
Substitution			138.38		20.582		< .001		.314		248.36	
Gate opening			136.98		13.994		< .001		.237		28.278	
Gate closing			120.89		13.686		< .001		.233		35.208	
**Difference in the effect between the groups**												
Condition		Calculation	ms		*F*(1,45)		*P*		η_p_^2^		BF_incl_	
Updating		TRIAL TYPE × AGE interaction	-4.47		0.1		.754		.002		0.293	
Substitution		TRIAL TYPE × RESPONSE × AGE interaction	67.82		12.984		< .001		.224		44.259	
Gate opening		GATE SWITCH (reference) × AGE interaction	12.18		0.648		.425		.014		0.38	
Gate closing		GATE SWITCH (comparison) × AGE interaction	-28.95		2.554		.117		.054		0.692	
**Difference between the groups in the size of the effect**												
		Calculation	ms	*t* (45)		*U*	*p*	*d*		*r*		BF_10_
RT difference	Updating	no-switch reference—no-switch comparison	-4.47	0.316		-	.754	0.092		-		0.302
	Substitution	(different no-switch reference—same no-switch reference)—(different no-switch comparison—same no-switch comparison)	67.82	-		428	< .001	-		.551		12.045
	Gate opening	switch reference—no-switch reference	12.18	-		328	.276	-		.188		0.409
	Gate closing	switch comparison—no-switch comparison	-28.95	-		230	.336	-		-.167		0.578
RT ratio	Updating	RT difference/(RT sum/2)	-0.03	-1.104		-	.275	-0.322		-		0.475
	Substitution	no-switch reference RT difference/(RT sum/2)–no-switch comparison RT difference/(RT sum/2)	0.08	2.757		-	.008	0.805		-		5.591
	Gate opening	RT difference/(RT sum/2)	0.01	-		327	.285	-		.185		0.401
	Gate closing	RT difference/(RT sum/2)	-0.07	-		173	.028	-		-.373		3.745

**Table 6:** A summary of the ERP statistical results regarding the differences in the subprocesses between the groups and between electrode sites.

**Difference in the effect between the groups (Main effect of AGE)**						
Condition	Time Window	*F*(1,45)	*P*	η_p_^2^	ε	BF_incl_
Updating	80–180 ms	0.756	.389	.017	-	0.312
	300–400 ms	1.609	.211	.035	-	0.522
	400–1,000 ms	5.818	.02	.114	-	2.541
Substitution	250–600 ms	0.258	.614	.006	-	0.285
	550–750 ms	3.601	.064	.074	-	1.134
Gate opening	150–250 ms	9.168	.004	.169	-	9.413
	300–500 ms	5.675	.021	.112	-	2.778
	500–700 ms	1.877	.177	.04	-	0.579
Gate closing	150–300 ms	0.058	.81	.001	-	0.307
	300–600 ms	0.153	.696	.003	-	0.286
**Difference in the effect between electrode sites (Main effect of ANTERIORITY)**						
Condition	Time Window	*F*(1,45)	*P*	η_p_^2^	ε	BF_incl_
Updating	80–180 ms	103.262	< .001	.696	.536	2.055×10^34^
	300–400 ms	22.762	< .001	.336	.608	1.714×10^9^
	400–1,000 ms	53.639	< .001	.544	.639	1.549×10^18^
Substitution	250–600 ms	2.853	.054	.06	.783	0.778
	550–750 ms	2.973	.053	.062	.704	0.694
Gate opening	150–250 ms	9.785	< .001	.179	.573	610.869
	300–500 ms	10.337	< .001	.187	.48	1757.509
	500–700 ms	11.4	< .001	.202	.611	11554.696
Gate closing	150–300 ms	19.183	< .001	.299	.676	7.503×10^7^
	300–600 ms	3.85	.027	.079	.633	2.729
**Difference in the effect between the groups depending on electrode site (AGE × ANTERIORITY interaction**						
Condition	Time Window	*F*(1,45)	*P*	η_p_^2^	ε	BF_incl_
Updating	80–180 ms	2.079	.142	.044	.536	0.573
	300–400 ms	2.36	.106	.05	.608	0.764
	400–1,000 ms	10.375	< .001	.187	.639	5485.974
Substitution	250–600 ms	4.457	.01	.09	.783	8.633
	550–750 ms	4.097	.018	.083	.704	5.361
Gate opening	150–250 ms	7.589	.002	.144	.573	257.032
	300–500 ms	7.568	.003	.144	.48	253.923
	500–700 ms	3.73	.032	.077	.611	3.577
Gate closing	150–300 ms	0.699	.502	.015	.676	0.122
	300–600 ms	1.809	.172	.039	.633	0.405

**Table 7:** Source differences between the younger and the older group. Regions, left (L) or right (R) hemisphere, number of significant voxels in the region as well as the MNI (x,y,z), t-value, and p-value of the peak difference voxel are shown.

			MNI coordinates				
Region	HM	*N*_*voxel*_	X	y	z	*t*	*p*
*Updating*							
400–1000 ms							
Lateral orbitofrontal gyrus	R	21	30.9	20.9	-26.2	3.786	0.0002
Insula	R	19	26.8	7.4	-25.7	3.639	0.0002
Inferior temporal gyrus	R	42	46.8	1.8	-48.3	4.235	0.0002
Middle temporal gyrus	R	47	50.6	4.9	-43.0	4.210	0.0002
Superior temporal gyrus	R	52	47.9	22.1	-27.5	4.169	0.0002
Temporal pole	R	33	37.8	25.0	-36.3	4.009	0.0002
Entorhinal cortex	R	28	27.0	-0.8	-39.5	3.709	0.0002
Fusiform gyrus	R	22	29.2	-3.7	-51.7	3.812	0.0002
*Gate opening*							
500–700 ms							
Rostral anterior cingulate	L	21	-1.4	14.2	-14.7	3.375	0.0004
	R	6	2.1	17.4	-12.1	3.296	0.0006
Lateral orbitofrontal gyrus	L	108	-21.1	9.9	-27.8	3.640	0.0004
Medial orbitofrontal gyrus	L	113	-14.8	7.6	-21.9	3.537	0.0004
	R	42	4.7	8.1	-20.2	3.549	0.0010
Precentral gyrus	L	9	-43.4	0.4	5.9	3.375	0.0008
Insula	L	83	-25.8	6.2	-21.0	3.607	0.0004
	R	7	2.1	7.8	-15.6	3.535	0.0008
Lingual gyrus	L	12	-15.6	-43.4	-14.2	3.643	0.0008
Inferior temporal gyrus	L	129	-30.5	8.5	-41.2	3.549	0.0004
Middle temporal gyrus	L	119	-53.6	-20.1	-14.0	3.532	0.0002
Superior temporal gyrus	L	111	-32.7	10.5	-26.0	3.461	0.0004
Temporal pole	L	27	-21.9	9.9	-32.0	3.627	0.0004
Transverse temporal gyrus	L	11	-33.0	-25.3	8.1	3.421	0.0002
Entorhinal cortex	L	27	-15.5	-16.7	-25.7	3.932	0.0004
Fusiform gyrus	L	113	-21.6	-38.3	-18.7	3.737	0.0006
Parahippocampal gyrus	L	61	-16.5	-25.0	-19.4	4.136	0.0004

### 3.1. Overall behavioural results

Overall accuracy was relatively high in both groups (*M* = 85.9% in the younger and *M* = 80.8% in the older group). The accuracy was somewhat lower in the older group than in the younger group, *U* = 154, *p* = .01, *r* = -0.442, BF_10_ = 4.858. This was due to the older group having a higher rate of misses than the younger group (5% vs. 3.1%); *U* = 422, *p* = .002, *r* = 0.529, BF_10_ = 9.387; while the evidence for a higher rate of incorrect responses in the older group was insufficient, *U* = 373.5, *p* = .039, *r* = 0.353, BF_10_ = 1.477 (11% in the younger vs. 14.1% in the older group).

The older participants were slower overall than the younger group (521.76 ms in the younger vs. 652.41 ms in the older group), *U* = 434.00, *p* < .001, *r* = 0.572, BF_10_ = 29.783.

### 3.2. Updating

**Fig 2:**
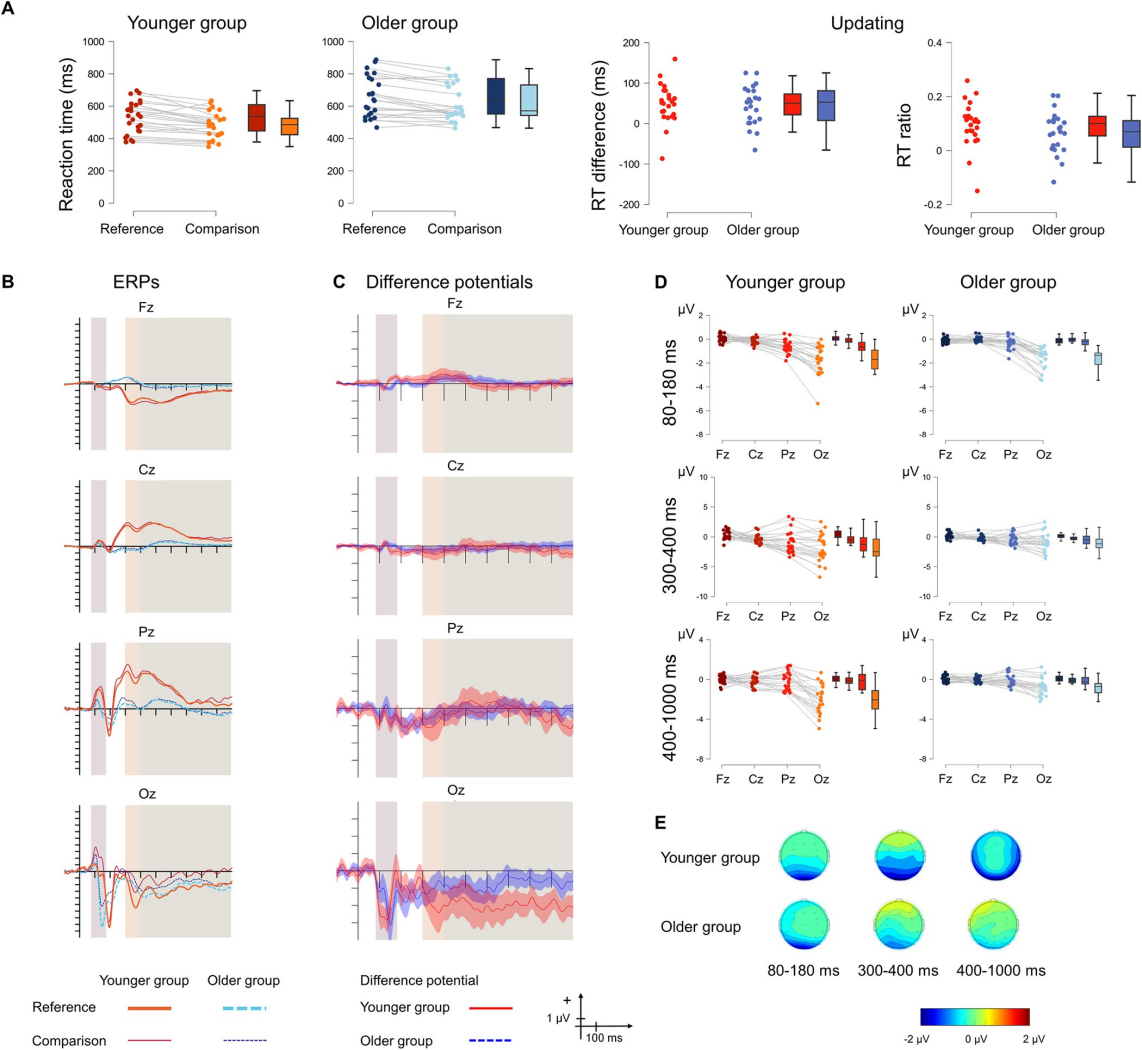
**A** Behavioural statistical results for *updating* in the younger (red) and the older (blue) groups as well as for the size of the effect (right panel). The darker shade shows the reference condition, and the lighter shade shows the comparison condition. **B** ERPs to the no-switch reference and no-switch comparison conditions and **C** Difference potentials with 95% CI intervals. Red continuous lines indicate the younger group, while blue dashed lines indicate the older group. For the ERPs the thick lines show the no-switch reference ERPs and the thin lines show the no-switch comparison ERPs. The shaded areas show the three time windows for analysis. **D** ERP statistical results for the difference in the updating effect between the two age groups for the 80–180 ms (top), 300–400 ms (middle) and 400–1,000 ms (bottom) time windows. Red colour indicates the younger group and blue colour indicates the older group. **E** Scalp distributions for the 80–180 ms (left), 300–400 ms (middle) and 400–1,000 ms (right) time windows for the younger (top) and the older (bottom) groups.

Fig 2 displays the behavioural and the ERP results.

#### 3.2.1. Behavioural results

As expected, the older participants were slower overall than the younger participants (639 ms vs. 506 ms). The *updating cost* was present (594 ms for reference vs. 548 ms for comparison trials), but there was no difference in the effect between the groups with moderate evidence for the exclusion of the interaction from the model. A comparison of the size of the effect between the two age groups confirmed that there was no difference either for the RT difference (48.72 ms in the younger and 44.25 ms in the older group) or the RT ratio (0.093 in the younger and 0.067 in the older group), although the evidence for the null hypothesis for the latter case was inconclusive.

#### 3.2.2. ERP results

In the 80–180 ms time window, there was moderate evidence for no contribution of the main effect of AGE GROUP to the model. The main effect of ANTERIORITY was significant. The amplitude measured at Oz was significantly more negative than those measured at Fz, Cz, and Pz (all *p* < .001). The amplitude measured at Pz was significantly more negative from those measured at both Fz and Cz (*p* = .002, and *p* = .009, respectively). The interaction was not significant but the evidence for excluding it from the model was insufficient.

In the 300–400 ms time window, there was insufficient evidence for excluding the main effect of AGE GROUP from the model. The main effect of ANTERIORITY was significant. The amplitude measured at Fz was significantly more positive than that measured at Cz, Pz, and Oz (all *p* < .001). The amplitude measured at Oz was significantly more negative than those measured at Cz and Pz (*p* < .001 and *p* = .002, respectively). The evidence for excluding the interaction from the model was also insufficient.

In the 400–1,000 ms time window, there was only anecdotal evidence for including the main effect of AGE GROUP in the model. The main effect of ANTERIORITY was significant. The amplitude measured at Oz was significantly more negative from those measured at Fz, Cz, and Pz (all *p* < .001). The interaction was also significant, and was caused by a significant difference in amplitude between the younger and the older group at Oz (*p* < .001), with the amplitude being more negative in the younger group.

#### 3.2.3. Source localisation results

**Fig 3:**
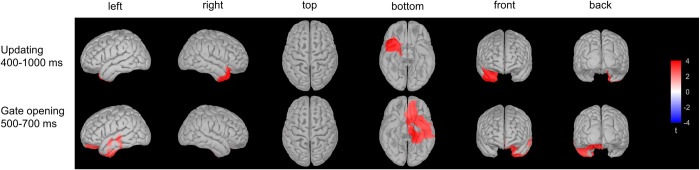
Source differences between the two age groups in *updating* (top) in the 400–1,000 ms time window and in *gate opening* (bottom) in the 500–700 ms time window.

Between-group differences were found only in the 400–1,000 ms time interval (Table 1 and Fig 3). Activation was larger in the younger group in the right hemisphere, in frontal (lateral orbitofrontal gyrus and insula) and temporal (inferior temporal gyrus, middle temporal gyrus, superior temporal gyrus, temporal pole, entorhinal cortex, fusiform gyrus) regions.

#### 3.2.4. Summary

Behavioural *updating* was present in both groups, but did not differ between them. The ERP results suggested a posterior distribution in all time windows for both groups. Furthermore, the two age groups differed in the late (400–1,000 ms) time window, where a longer duration posterior negativity was observed in the younger group. In the same late time window, source localisation showed larger differences in brain activation for the younger group in the right temporal cortex and right prefrontal cortex.

### 3.3. Substitution

**Fig 4:**
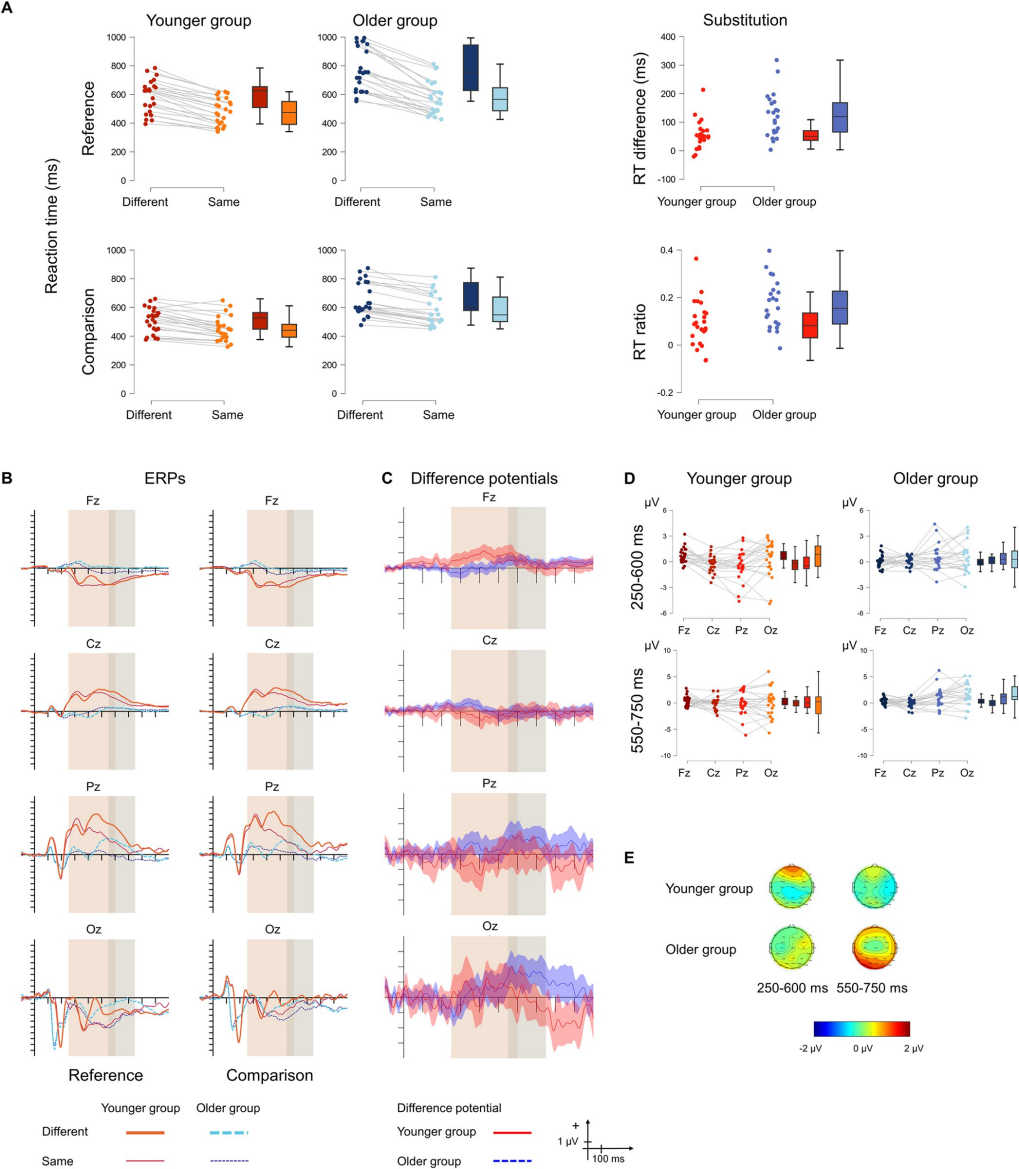
**A** Behavioural statistical results for *substitution* in the younger (red) and the older (blue) groups as well as for the size of the effect (right panel). The two panels on the left show the no-switch reference trials, and the two panels on the right show the no-switch comparison trials. The darker shade shows the “different”, and the lighter shade shows the “same” condition. **B** ERPs to the no-switch reference (left) and no-switch comparison (right) conditions and **C** Difference potentials with 95% CI intervals. Red continuous lines indicate the younger group, while blue dashed lines indicate the older group. For the ERPs the thick lines show the “different” ERPs, and the thin lines show the “same” ERPs. The shaded areas show the two time windows for analysis. **D** ERP statistical results for the difference in the substitution effect between the two age groups for the 250–600 ms (top) and 550–750 ms (bottom) time windows. Red colour indicates the younger group and blue colour indicates the older group. **E** Scalp distributions for the 250–600 ms (left) and 550–750 ms (right) time windows for the younger (top) and the older (bottom) groups.

Fig 4 displays the behavioural and the ERP results.

#### 3.3.1. Behavioural results

The older group was slower overall than the younger group (646 ms vs. 508 ms). *Substitution* was significant as seen in the TRIAL TYPE × RESPONSE interaction. While participants were slower overall for both different no-switch reference stimuli compared to same no-switch reference stimuli (682 ms vs. 526 ms, *p* < .001); and different no-switch comparison compared to same no-switch comparison stimuli (580 ms vs. 513 ms, *p* < .001); there was a significant difference between no-switch reference and comparison stimuli in the case of stimuli that required a “different” response (682 ms vs. 580 ms, *p* < .001); but not in the case of stimuli with the “same” response (526 ms vs. 513 ms, *p* = .721). There was also a significant difference in *substitution* between the groups as shown by the triple interaction. The older group was slower overall than the younger group, and the difference was significant in the following conditions: 1) different no-switch reference stimuli, 592 ms in the younger and 775 ms in the older group, *p* < .001; 2) different no-switch comparison stimuli, 510 ms in the younger and 653 ms in the older group, *p* = .001; and 3) same no-switch comparison stimuli, 451 ms in the younger and 578 ms in the older group, *p* = .006; but 4) the groups did not differ for the same no-switch reference stimuli, 477 ms in the younger and 577 ms in the older group, *p* = .077. There was moderate evidence for excluding the TRIAL TYPE × AGE GROUP interaction from the model. A direct comparison of the size of the substitution effect showed that the effect was larger in the older than in the younger group for both RT difference (123 ms vs. 55 ms, *p* < .001) and RT ratio (0.169 vs. 0.09, p = .008).

#### 3.3.2. ERP results

In the 250–600 ms time window, there was a tendency for a main effect of ANTERIORITY, but the evidence for its contribution to the model was inconclusive. The absence of a main effect of AGE GROUP was supported by moderate evidence for excluding the effect from the model. The interaction was significant and due to the mean amplitude measured at Fz being significantly more positive than that measured at Cz (.029) and Pz (*p* = .007) in the younger group. The evidence for including the interaction in the model was moderate.

In the 550–750 ms time window, there was a tendency for a main effect of both AGE GROUP and ANTERIORITY, but the evidence for including either in the model was insufficient. The interaction was significant with moderate evidence for its contribution to the model: there was an anteriority effect only in the older group, in which the amplitude measured at Oz was significantly more positive than that measured at Cz (*p* = .002). The younger and the older group differed significantly at the Oz electrode site (*p* = .034), with the amplitude being more positive in the older compared to the younger group.

#### 3.3.3. Source localisation results

There were no source differences between the groups.

#### 3.3.4. Summary

The behavioural results showed the presence of *substitution* in both age groups, with a larger effect observed in the older group. The ERP results indicated a difference between the groups in both time windows, which difference was dependent on the distribution of the effect. In the 250–600 ms time window, the effect was observable in the younger group and had a frontal distribution, while in the 550–750 ms time window, it was observable in the older group and had an occipital maximum.

### 3.4. Gate opening

**Fig 5:**
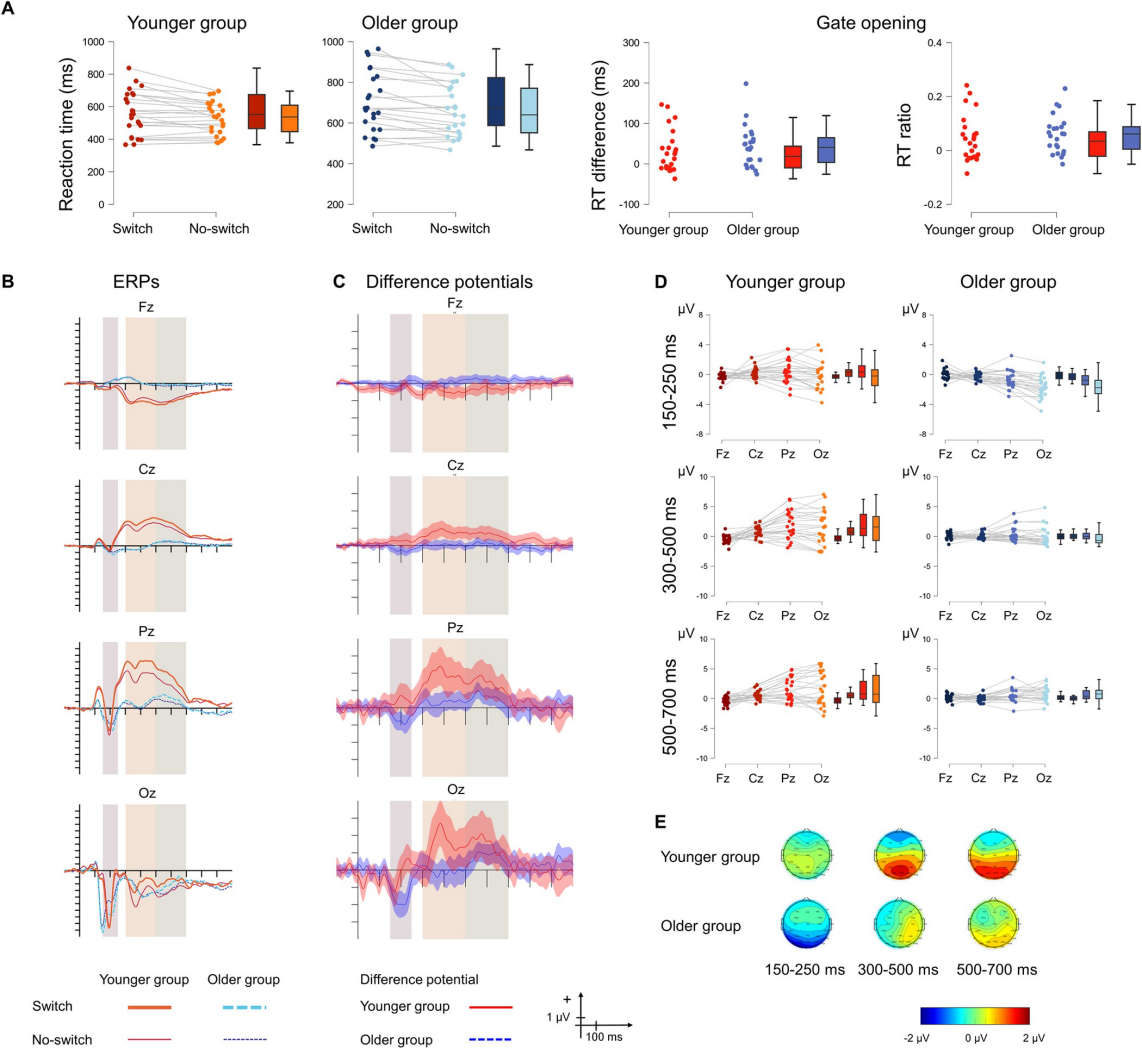
**A** Behavioural statistical results for *gate opening* in the younger (red) and the older (blue) groups as well as for the size of the effect (right panel). The darker shade shows the switch reference condition, and the lighter shade shows the no-switch reference condition. **B** ERPs to the switch reference and no-switch reference conditions and **C** Difference potentials with 95% CI intervals. Red continuous lines indicate the younger group, while blue dashed lines indicate the older group. For the ERPs the thick lines show the switch reference ERPs, and the thin lines show the no-switch reference ERPs. The shaded areas show the three time windows for analysis. **D** ERP statistical results for the difference in the gate opening effect between the two age groups for the 150–250 ms (top), 300–500 ms (middle) and 500–700 ms (bottom) time windows. Red colour indicates the younger group and blue colour indicates the older group. **E** Scalp distributions for the 150–250 ms (left), 300–500 ms (middle) and 500–700 ms (right) time windows for the younger (top) and the older (bottom) groups.

Fig 5 displays the behavioural and the ERP results.

#### 3.4.1 Behavioural results

The older group was slower overall than the younger group (682 ms vs. 545 ms). *Gate opening* was indicated by switch reference trials being slower than no-switch reference trials (630 ms vs. 594 ms). The GATE SWITCH × AGE interaction was not significant with inconclusive evidence regarding its contribution to the model. The direct comparison of the size of the effect supported this result, which was 42.54 ms in the older and 30.36 ms in the younger group for the RT difference, and 0.059 in the older and 0.045 in the younger group for the RT ratio, although the evidence for the null hypothesis was insufficient.

#### 3.4.2. ERP results

In the 150–250 ms time window, there was a main effect of AGE GROUP. The measured amplitude was overall more negative in the older group. The main effect of ANTERIORITY was also significant. The amplitude measured at Pz was significantly more negative than those measured at Fz and Cz (*p* < .001 and *p* = .029, respectively). The amplitude measured at Oz was significantly more negative than that measured at Fz (*p* = .002). The interaction was significant. The anteriority effect was only seen in the older group with the amplitude measured at Oz being significantly more negative than those measured at Fz, Cz, and Pz (*p* < .001, *p* < .001, and *p* = .013, respectively). The two age groups differed significantly at the Pz (*p* = .03) and Oz (*p* < .001), with the amplitude measured in the older group being more negative.

In the 300–500 ms time window, the overall amplitude in the younger group was more positive than that measured in the older group, and the main effect of AGE GROUP was significant, but the evidence for its contribution to the model was insufficient. The main effect of ANTERIORITY was significant. The amplitude measured at Oz was significantly more positive than those measured at Fz, Cz, and Pz (all *p* < .001). The interaction was significant. The anteriority effect was observable only in the younger group, in which the amplitudes measured at Cz, Pz, and Oz were more positive than that measured at Fz (*p* = .015, *p* < .001, and *p* < .001, respectively). The amplitude measured at Pz was more positive than that measured at Cz (*p* = .039). The amplitudes measured in the younger group at the Pz and the Cz electrode sites were more positive than those measured in the older group (*p* = .03 and *p* = .033, respectively).

In the 500–700 ms time window, there was no main effect of AGE GROUP, although the evidence for its exclusion from the model was inconclusive. The main effect of ANTERIORITY was significant. The amplitude measured at Fz was significantly smaller than those measured at Pz and Oz (both *p* < .001). The amplitude measured at Cz was also significantly smaller than those measured at Pz and Oz (*p* = .042 and *p* = .008, respectively). The interaction was significant, with moderate evidence for including it in the model. In the younger group, the amplitude measured at Fz was significantly smaller than those measured at Pz (both *p* < .001).

#### 3.4.3 Source localisation results

There were source differences between the age groups in the 500–700 ms time interval (Table 7 and Fig 3). Activation was larger in the younger compared to the older group in the left hemisphere, mainly in frontal (orbitofrontal gyrus, precentral gyrus, insula, rostral anterior cingulate) and temporal (inferior temporal gyrus, middle temporal gyrus, superior temporal gyrus, temporal pole, transverse temporal gyrus, entorhinal cortex, fusiform gyrus, parahippocampal gyrus) regions. There was also larger activation in the younger compared to the older group in the right hemisphere, in frontal regions (medial orbitofrontal cortex, insula, rostral anterior cingulate).

#### 3.4.4. Summary

Although the behavioural results did not show a difference in *gate opening* between the age groups, the ERP results suggest that the brain processes differed between them. The older group had more negative amplitudes in the parieto-occipital regions than the younger group in the early time window, while the younger group showed a difference in the distribution of the effect in the two later windows, with the mean amplitude being largest over the parietal region and being larger than that of the older group in the middle time window. Additionally, source localisation analysis revealed larger differences in brain activation in the younger group only during the last time window (500–700 ms), specifically over the left and right frontal regions and the left temporal regions.

### 3.5. Gate closing

**Fig 6:**
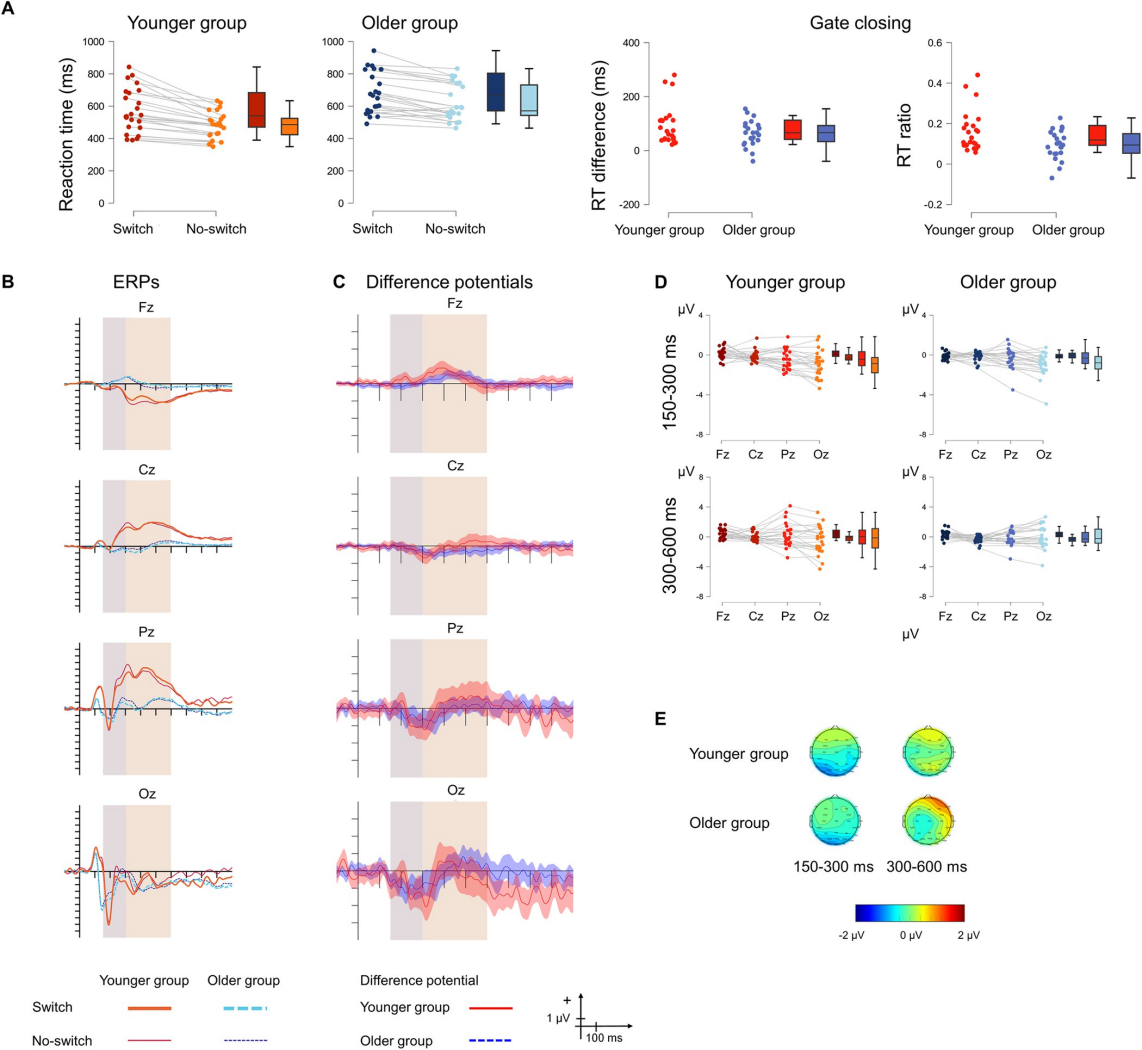
**A** Behavioural statistical results for *gate closing* in the younger (red) and the older (blue) groups as well as for the size of the effect (right panel). The darker shade shows the switch comparison condition, and the lighter shade shows the no-switch comparison condition. **B** ERPs to the switch comparison and no-switch comparison conditions and **C** Difference potentials with 95% CI intervals. Red continuous lines indicate the younger group, while blue dashed lines indicate the older group. For the ERPs the thick lines show the switch comparison ERPs, and the thin lines show the no-switch comparison ERPs. The shaded areas show the two time windows for analysis. **D** ERP statistical results for the difference in *gate closing* between the two age groups for the 150–300 ms (top) and 300–600 ms (bottom) time windows. Red colour indicates the younger group and blue colour indicates the older group. **E** Scalp distributions for the 150–300 ms (left) and 300–600 ms (right) time windows for the younger (top) and the older (bottom) groups.

Fig 6 displays the behavioural and the ERP results.

#### 3.5.1. Behavioural results

The older group was slower overall than the younger group, 648 ms vs. 527 ms. *Gate closing* was also present overall, 625 ms for switch comparison compared to 548 ms for no-switch comparison stimuli. There was no difference between the groups with insufficient evidence for excluding the interaction from the model. The direct comparison of the size of *gate closing* between the older (62.88 ms) and the younger (91.83 ms) also showed no significant difference for the RT difference, with insufficient evidence for the null hypothesis. Regarding the RT ratio, a smaller gate closing effect is observed in the older (0.095) compared to the younger group (0.161). Of note, the difference seems to be driven by three outlier values in the younger group, and the removal of the outliers leads to only a trend in the difference, *t*(45) = -1.745, *p* = 0.088, *Cohen’s d* = -0.527, BF_10_ = 0.995.

#### 3.5.2. ERP results

In the 150–300 ms time window, there was moderate evidence for excluding the main effect of AGE GROUP from the model. The main effect of ANTERIORITY was significant. The amplitude measured at Oz was significantly more negative than those measured at Fz, Cz, and Pz (*p* < .001, *p* < .001, and *p* = .001, respectively). The amplitude measured at Pz was significantly more negative than that measured at Fz (*p* = .007). There was moderate evidence for no contribution of the interaction to the model.

In the 300–600 ms time window, there was moderate evidence for the exclusion of the main effect of AGE GROUP. There was insufficient evidence for the contribution of the main effect of ANTERIORITY to the model. There was also insufficient evidence for the exclusion of the interaction.

#### 3.5.3. Source localisation results

There were no source differences between the groups.

#### 3.5.4. Summary

We obtained insufficient evidence for any behavioural differences in the *gate closing* between the two groups as a potential significant difference was likely influenced by outlier values. No ERP differences were found between the age groups, with the effect showing an occipital maximum only in the early time window.

## 4. Discussion

Our study employed a data-based approach to explore the electrophysiological correlates of the working memory subprocesses revealed by the reference-back paradigm. Given the absence of prior results, we specifically investigated which subprocesses differ in younger and older adults, and how.

We replicated the experimental design of the reference-back paradigm initially introduced by Rac-Lubashevsky and Kessler [2] and obtained similar behavioural results. We observed significant costs for all four subprocesses we measured: *updating*, *substitution*, *gate opening* and *gate closing*. These results, consistent with others [3, 9], confirmed the robustness of these subprocesses. Beyond the usual age-related slowing in reaction time, we found differences between the age groups only in *substitution*. This cost of updating working memory with new information was larger in older compared to younger adults. As the RT ratio method eliminates age-related slowing, we might conclude from behavioural results that *substitution* is the most vulnerable among the examined subprocesses in the elderly. However, the ERP data provides a deeper insight into what may underlie these subprocesses.

For *updating*, the cluster-based permutation analysis and visual inspection suggested three negative ERP components in both groups, which showed the processing difference between reference and comparison trials in the no-switch trials. Although we could say that this pattern in itself characterizes the *updating* process, it is worth examining what other processes may contribute to its development. Can we assume there are differences between the two trial types other than the *updating* process, which are expressed in individual components and do not necessarily characterize *updating*?

The negativity between 80–180 ms had an occipital maximum. It most likely indicates differences in visual processing and not *updating* per se. Firstly, the stimuli were physically different: while the frame colour was red in the reference trials, it was blue in the comparison trials. Secondly, the red frame was a prompt for working memory updating, and attentional resources may have been better allocated to it. This could have been indicated by the occipital N1 component which reflects a discrimination effect in the attended area [55, 56]. The two age groups did not show differences in this processing stage: neither in the size of the amplitude, nor in its distribution, nor in the source of the component.

The negativity observed between 300–400 ms also showed an occipital maximum, and demonstrated anteriority, but did not manifest any other significant effects. The component may belong to the N2 family and can reflect selective attention [57]. Given that the reference and comparison trials have different significance in the task, it is reasonable to assume that the attention given to them is not entirely the same, as the greater significance of the reference trials was reflected in its larger amplitude.

Typically, within this time window, we would expect the P3b component to characterize the updating of working memory content, resulting in a positive component seen parietally [15, 58]. Using the reference-back paradigm, Rac-Lubashevsky and Kessler [13] could not confirm that there would be a connection between P3b and WM updating; instead, they concluded that P3b reflected target categorization process. In contrast to Rac-Lubashevsky and Kessler, we analysed the difference curves, isolating the neural processes that differ in the two trial types; and consequently, characterize the *updating* process. In the older age group, the original curve lacks a prominent P3b, consistent with the idea that this component may not necessarily emerge in more challenging tasks for them [59]. Therefore, it is not surprising that the difference curve does not reveal this component in their case either. While we observed the P3b component in the original curves for younger participants, it was absent in the difference curve. Accordingly, we could conclude that the P3b component does not play a role in updating working memory content aligning with the notion that the component may indicate other processes, such as the closure of cognitive epochs [60, 61]; stimulus-response link reactivation [62, 63]; response facilitation [64]; or decision making [65, 66]. However, whereas in Rac-Lubashevsky and Kessler’s model *updating cost* entails the updating of working memory content with both new and old information, other studies define updating as the incorporation of new information, akin to *substitution* in the current model. This implies that we should evaluate the theory that P3b reflects working memory updating when discussing the *substitution* results.

Finally, we found a significant negative shift in the younger but not the older group between 400 and 1,000 ms occipitally. In the two age groups, there are differences again in the course of the original curves: in older adults, there is hardly any difference between the two trial types, indicating less differentiation between the tasks in this later stage; whereas in younger adults, the curve returns to baseline at about 600 ms in comparison trials, while in reference trials, when updating is required, there is a noticeable large negativity during the whole epoch. Source analysis also confirmed age-group differences, as larger activity was found in the right frontal and temporal areas in the younger compared to the older adults. Similar late posterior negativity was found when the memory task required the binding of items with contextual information specifying the given episode [67].

In now summarizing the *updating* results, we suggest that the occipital negativity between 300 and 1,000 ms characterizes this process in younger adults, while it appears that older adults do not distinctly differentiate between trials that require both matching and updating, and trials that require only matching.

While the *updating cost* encompasses both the updating of working memory with new and old information, *substitution* represents a more specific process, indicating solely the updating of working memory with new information. This is calculated through a double subtraction, where we contrast the matching components within the no-switch trials between reference and comparison trials. Cluster analysis showed two significant time windows after this subtraction: a component between 250–600 ms for the younger group, and another one between 550–750 ms for the older adults. In younger adults, the positivity between 250–600 ms showed a frontal maximum, while in older adults, the positivity between 550–750 ms had a posterior maximum. Overall, in both groups, the electrophysiological signal of updating with new information differed from the pattern observed during *updating*, and these components can be compared with the P3b obtained in other experiments. Although the P3b component typically shows a later latency in the older group; its distribution is parietal in younger individuals, whereas in older adults, it may show a more uniform distribution, with a smaller parietal peak and the emergence of a frontal maximum as well [e.g., 18, 19]. However, the pattern observed here suggests different underlying processes between the two age groups, which is further supported by the sLORETA results. While the group differences of the sources were not significant, the group results, shown in the S5 File though not discussed in detail here, indicated a greater involvement of frontal sources in younger adults and posterior sources in older adults. Our within-group sLORETA analysis highlighted areas showing higher activity difference between the conditions relative to the average activity difference across the entire scalp. However, the within-group brain activity differences could still be similar across groups, e.g., the larger difference in the parietal region could be similar magnitude in the two groups, but of different magnitude when compared to other regions within the group. Therefore, it is possible that although different sources are found when analysing age groups separately, we do not obtain significant results when comparing groups in a whole-brain analysis. Despite this methodological limitation, this result suggests a significant difference between the two age groups in one of the subprocesses of working memory as different brain regions are activated at different time intervals, which may be a key to why the working memory of older adults is poorer.

On the one hand the frontal area is thought to play a role in the active manipulation of information, acting as an attentional filter to determine which information will be selected for maintenance in working memory; while on the other hand the parietal cortex is involved in the actual maintenance of that information [68]. One possible reason why older adults activate the frontal areas less than younger ones, may be because this region reacts most sensitively to aging in terms of both structural and functional changes [69]; and therefore, another region takes over its role, which has a better preserved structural integrity. However, this raises the question of how this aligns with findings showing over-recruitment of frontal areas in older adults compared to younger adults when performing high-demand tasks [e.g., 27, 70]. While we cannot provide a definitive answer, it is possible that in certain cases, the two age groups employ different strategies to solve the same task, utilizing distinct processes [e.g., 12]. This could explain why different brain areas are activated and why frontal compensation is not always observed. Additionally, we should note that this time window occurs after the response has already been made in younger adults, whereas response organization is still ongoing in older adults. Thus, it is possible that the differences in this range are not solely due to working memory-related processes.

According to the PBWM model, a gating mechanism enables the updating of working memory, and also ensures the protection of its content. *Gate opening* makes possible the entry of information into working memory and allows for its updating to occur. We found three components that significantly emerged from the EEG: two in the younger group, and two in the older group, with one component overlapping and present in both age groups. When analysing *gate opening*, it is important to consider that this reflects the difference between reference switch and reference no-switch trials. Consequently, two important parameters do not equalize. One being that in the trial preceding the switch trials, the frame colour differs from the colour of the current frame, while in no-switch trials, the colour of the previous and currently viewed frame is the same. The other being their frequency difference: the probability of switch is 0.25, while the probability of no-switch trials is 0.75. Both are parameters that may influence the emergence of the ERP components.

The first component we observed is a negativity over the posterior regions between 150–250 ms. In this range, various attentional effects are present, suggesting the possibility of selection negativity which distinctly influenced by the differential occurrence probabilities of the trial types [57, 71, 72]. Hence, it is possible that the emergence of the component was due to the differences between the two trial types and does not inherently characterise the *gate opening* process. This component had a larger amplitude in the older than the younger group, which suggests they were more involved in the selective analysis of the visual input.

The second component emerged between 300–500 ms and reached its positive maximum over the posterior areas. Given the similarity with the P3b component in oddball studies, differing probabilities may also influence this component [73, 74]. This component may significantly contribute to the context updating-related P3b observed when the separation of subprocesses is omitted. Younger adults had a larger amplitude over the centro-parietal regions than the older ones, indicating emphasized processes in the former group.

The third component between 500–700 ms was also a positivity and had a parieto-occipital maximum. In the literature, the P600 component is known to occur over the posterior areas within this time window, encompassing both linguistic processes and executive functions [75]. It has also been found that the basal ganglia, which play a crucial role in gate opening in the PBWM model, modulate the P600 [76]. Alternatively, P600 belongs to the P3 family and is connected to cortical reorientation [77]. No age-related differences were found in the amplitude; however, larger activation was observed in extensive left-lateralized areas, mainly the orbitofrontal cortex and the lateral and medial temporal lobes in the younger group compared to the older one, in areas which have a role in: mapping contingencies [78]; object recognition [79]; consolidation and retrieval [80, 81], respectively.

In summary, in *gate opening* not all conditions are balanced, so we cannot be certain that after the subtractions, we only see the effect of the subprocess emerging in the EEG. Nevertheless, we found age-related differences in all three evoked components, suggesting that this subprocess may operate with varying efficiency in the younger and older groups.

While it is important to be able to update the contents of working memory with new information, it is equally essential for efficient functioning to be able to protect it by ensuring that it remains intact against the influx of new information from external or internal sources. The protection of existing contents is made possible by *gate closing*. We found two components, similar in both groups, that characterized this process: a negativity above the occipital electrodes between 150–300 ms; and a frontal positivity between 300–600 ms. Considering that, as with *gate opening*, here also exist differences which are not eliminated by subtraction–in the comparison switch trial, the frame colour differs from the colour of the frame seen in the previous trial, while in the comparison no-switch trial, it matches; and the former occurs with a probability of 0.25, while the latter with 0.75 –so we can assume similar processes to those previously described for *gate opening*. However, the two age-groups showed no difference in the amplitude, scalp distribution or the source of this component.

The positivity between 300–600 ms had a frontal maximum in both age-groups; however, the cluster-based permutation analysis showed a significant cluster only in the older group. This is interesting for two reasons. Firstly, the involvement of frontal areas suggests that *gate closing* may be an active process, consistent with Nir-Cohen et al.’s [11] conception. Secondly, a common issue among older adults is their decreased ability to protect the contents of their working memory; so, they are very sensitive to the interference of task-irrelevant stimuli [e.g., 82, 83]. We might assume that this phenomenon is connected to the *gate closing* process as irrelevant information can intrude if the *gate closing* is not effective enough. By contrast, we did not find any differences between younger and older adults in either the amplitude or the source of this component.

Summarizing the *gate closing* results, this subprocess was characterized by a posterior negativity between 150–300 ms and a frontal positivity between 300–600 ms, neither of which was sensitive to aging.

A limitation of the study is that the sLORETA method is not suitable for identifying activity in the basal ganglia, so not all sources that are part of the PBWM model can be identified. This is the reason why we remained at a descriptive level for source localization, as our EEG data can only provide indicative information in this regard.

## 5. Summary

In this study our goal was to describe, for the first time, which ERP components are involved and how in the working memory subprocesses that can be disentangled using the reference back paradigm, as well as how aging affects these subprocesses. *Updating* was characterized by occipital negative components between 80–180 ms, 300–400 ms and 400–1,000 ms, with only the latter showing age-related differences. When analysing *substitution*, we observed a frontal positivity between 250–600 ms in younger adults, and a posterior positivity between 550–750 ms in older adults. In *gate opening* three parieto-occipital components emerged: a negativity between 150–250 ms, a positivity between 300–500 ms, and a positivity between 500–700 ms, all of which showed age-related differences. Finally, *gate closing* was characterised by an occipital negativity between 150–300 ms and a frontal positivity between 300–600 ms, neither of which changed between the two age groups.

Based on the above, we can conclude that the process of protecting information, i.e., *gate closing*, does not change with aging, contrary to what we might assume from older adults’ sensitivity to interference and inability to properly inhibit irrelevant information. Instead, *gate opening* is the process that is sensitive to age-related effects, and this is likely to be what leads to the different brain activity observed between the two age groups during *substitution*, i.e., the updating of working memory with new information.

## Supporting information

S1 File Error rate data and statistical analysis. (XLSX)

S2 File Reaction time data and statistical analysis. (XLSX)

S3 File Cluster-based permutation analysis results. (PDF)

S4 File Mean amplitude data and statistical analysis. (XLSX)

S5 File Within-group source localisation analysis results. (PDF)
