# Supplementary material for: Decomposing working memory subprocesses with the reference-back paradigm: Event-related potentials and age-related differences
Source: PLoS One. 2024 Dec 4;19(12):e0307351. doi: 10.1371/journal.pone.0307351 (PMC11616816; doi:10.1371/journal.pone.0307351)
Supplement: S3 File — (PDF) [file pone.0307351.s003.pdf]

Gaál, Z. A., Nagy, B., Czigler, I., Csizmadia, P., Petró, B., & Kojouharova, P. Age-related differences in working memory subprocesses decomposed by the reference-back paradigm

Cluster-based permutation *t*-test analysis

The values for the cluster-based permutation *t*-tests in our analysis are shown in Table 1 and displayed on Figure 1. Note that a replication of this analysis should result in similar but not identical values because of the analysis relies on permutations.

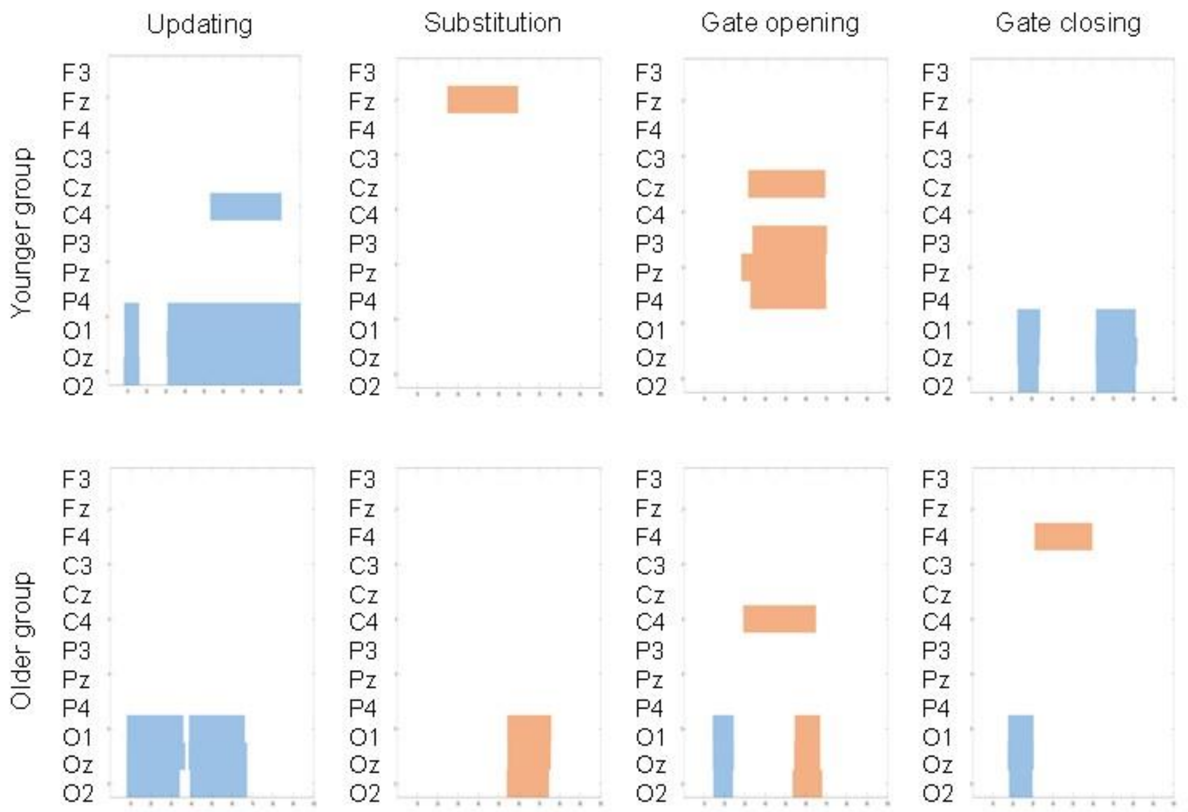

Figure 1. Positive (orange) and negative (blue) clusters found along the midline in the younger and in the older group for each condition. The x axis shows the time points from 0 to 1000 ms in 100 ms steps.

Table 1. A summary of the cluster-based permutation *t*-test results.

Effect	Group	sign	electrode	start (ms)	end (ms)	t_{max}	*p*
Updating	younger	negative	O2	79	161	-1276.37	0.0344
			OZ	83	159	-1276.37	0.0344
			O1	82	159	-1276.37	0.0344
		negative	O1	308	1000	-9511.85	0
			OZ	304	1000	-9511.85	0
			O2	307	1000	-9511.85	0
		negative	C4	531	902	-1099.9	0.0418
	older	negative	O1	81	358	-2903.71	0.002
			OZ	80	367	-2903.71	0.002
			O2	80	340	-2903.71	0.002
		negative	O1	386	660	-2434.44	0.0054
			OZ	384	671	-2434.44	0.0054
			O2	391	671	-2434.44	0.0054
Substitution	younger	positive	FZ	247	593	1143.353	0.0148
	older	positive	O1	541	756	1909.142	0.0024
			OZ	542	754	1909.142	0.0024
			O2	539	745	1909.142	0.0024
Gate opening	younger	positive	P3	336	701	1084.119	0.0334
			PZ	281	696	1365.046	0.0218
			P4	326	698	1137.748	0.0308
			CZ	315	693	1323.654	0.0224
	older	negative	O1	141	243	-1429.64	0.005
			OZ	142	244	-1429.64	0.005
			O2	144	240	-1429.64	0.005
		positive	O1	544	668	1211.896	0.0102
			OZ	541	668	1211.896	0.0102
			O2	533	678	1211.896	0.0102
		positive	C4	291	647	1138.516	0.0142
Gate closing	younger	negative	O1	228	340	-1124.19	0.021
			OZ	228	337	-1124.19	0.021
			O2	231	333	-1124.19	0.021
		negative	O1	615	809	-1817.43	0.0062
			OZ	613	817	-1817.43	0.0062
			O2	615	809	-1817.43	0.0062
	older	negative	O1	175	301	-1096.87	0.0214
			OZ	173	302	-1096.87	0.0214
			O2	180	296	-1096.87	0.0214
		positive	F4	307	594	1055.873	0.0234
