# Supplementary material for: Decomposing working memory subprocesses with the reference-back paradigm: Event-related potentials and age-related differences
Source: PLoS One. 2024 Dec 4;19(12):e0307351. doi: 10.1371/journal.pone.0307351 (PMC11616816; doi:10.1371/journal.pone.0307351)
Supplement: S5 File — (PDF) [file pone.0307351.s005.pdf]

Gaál, Z. A., Nagy, B., Czigler, I., Csizmadia, P., Petró, B., & Kojouharova, P. (submitted) Age-related differences in working memory subprocesses decomposed by the reference-back paradigm

Source localisation (within groups)

For each subject and each effect the sources for the respective ERPs were estimated, their difference computed, then normalized to baseline and flattened. The differences were compared to 0 with parametric one-sample $\chi 2$ -tests for unconstrained sources for each group, each effect, and each previously selected time window separately. This analysis shows when and where there are differences between the two conditions within each age group. The differences were reported as significant the Bonferroni corrected alpha level (alpha = .001) was exceeded, however, one of the possible outcomes is that all voxels show significant differences as the parametric one-sample $\chi 2$-test is a very sensitive test (Tadel et al., 2016). To introduce further restrictions and isolate the areas with greatest difference, for each time interval we determined the voxels for which the $\chi 2$-test values were above the mean plus two standard deviations as well as the regions that had at least 20 voxels with such values. The results are reported based on these constraints, summarized in Table 1, and displayed on Figure 1 (updating), Figure 2 (substitution), Figure 3 (gate opening), and Figure 4 (gate closing).

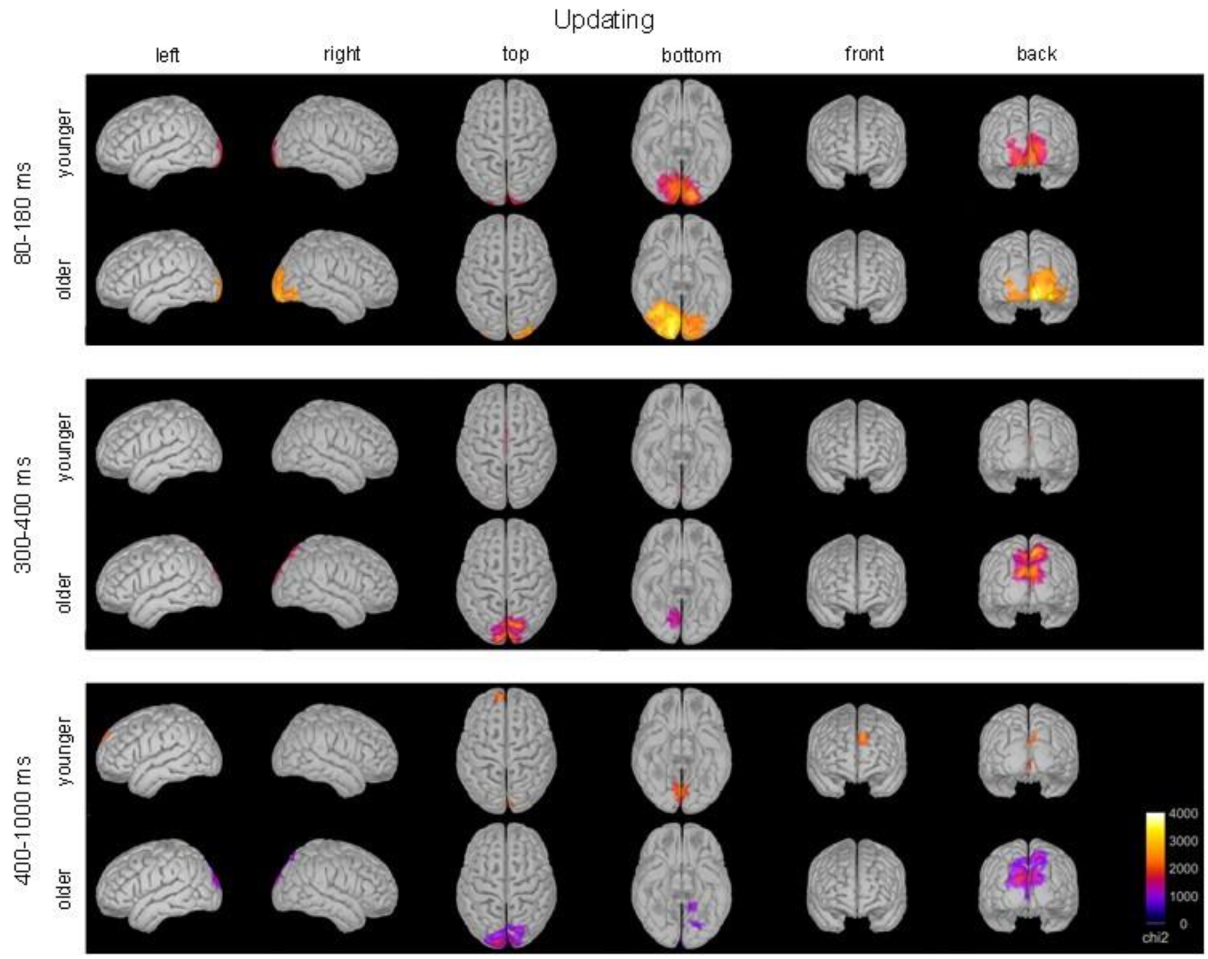

Figure 1. Largest source differences for the updating effect in each time window within the age groups.

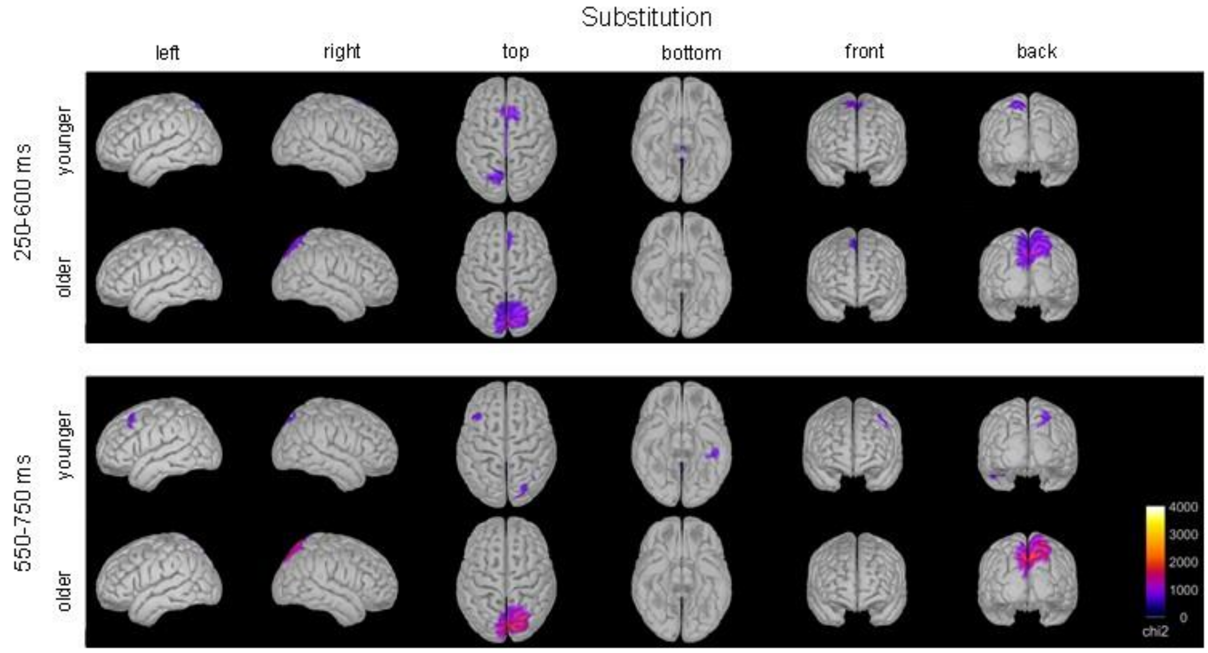

Figure 2. Largest source differences for the substitution effect in each time window within the age groups.

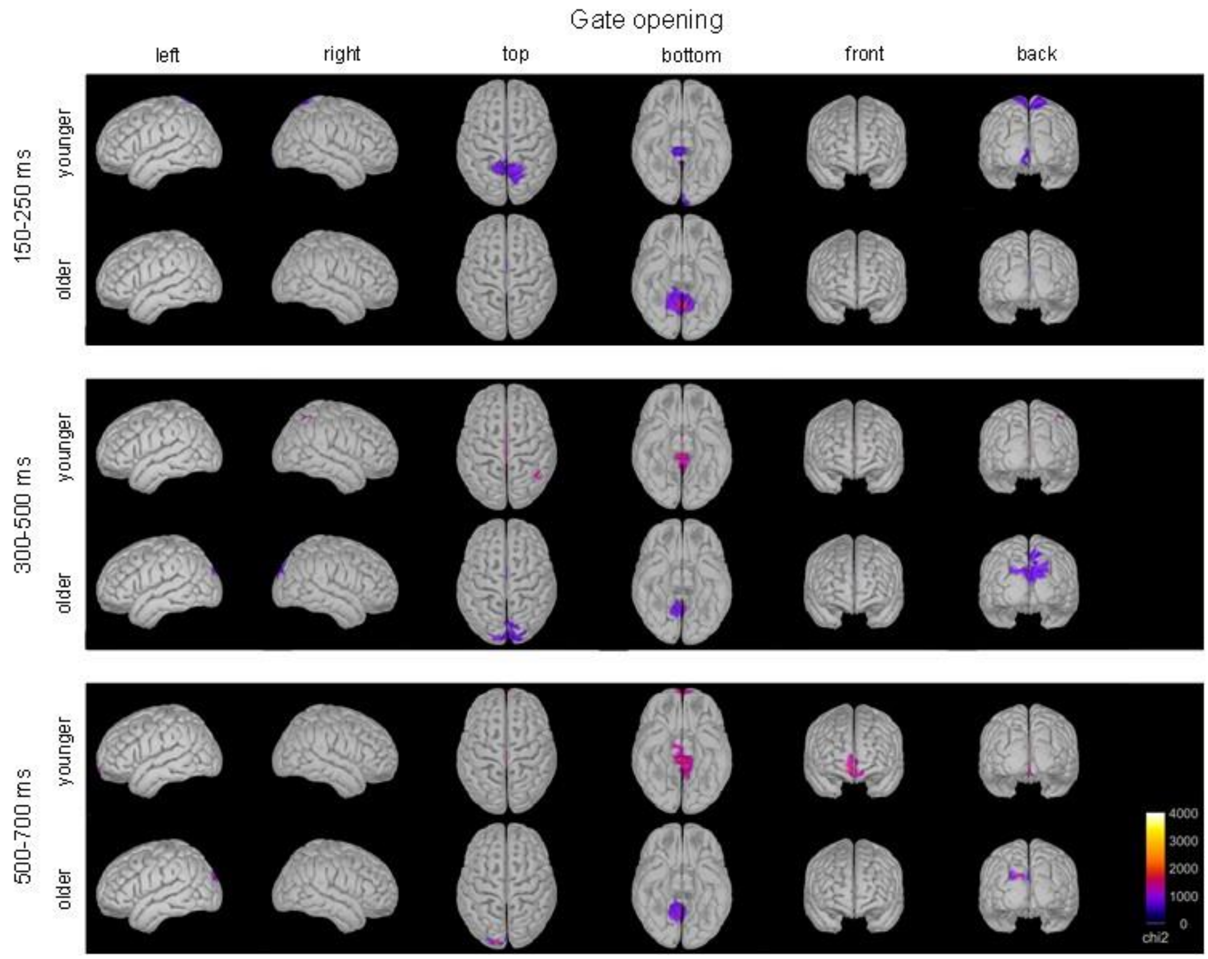

Figure 3. Largest source differences for the gate opening effect in each time window within the age groups.

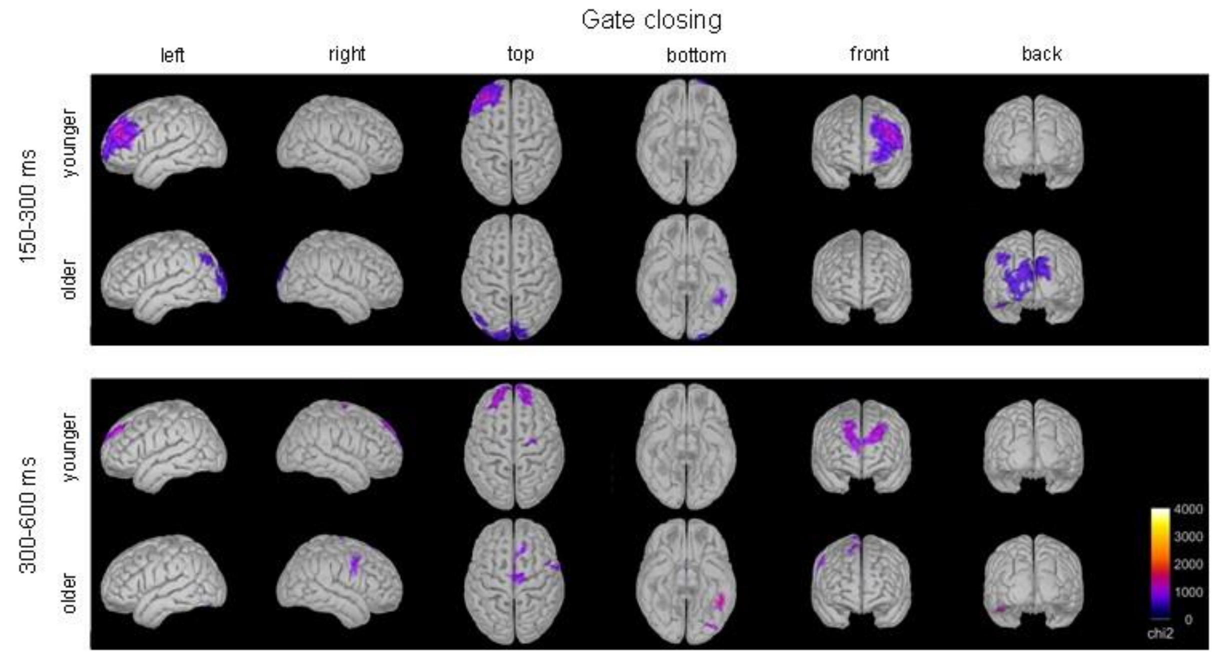

Figure 4. Largest source differences for the gate closing effect in each time window within the age groups.

Table 1. A summary of the largest source difference for each condition and each time window within the younger and within the older group. HM indicates left (L) or right (R) hemisphere, N shows the number of voxels passing the threshold within a region, and x, y, and z are the MNI coordinates of the voxel with peak χ^2 value.

				MNI coordinates			
Region	HM	Group	N_{voxel}	x	y	z	χ^2
Updating							
80-180 ms							
Cuneus	L	younger	59	1.2	-76.6	14.0	5144.7
		older	31	0.2	-86.6	-1.8	2839.9
	R	younger	48	2.2	-73.6	13.7	3476.9
		older	35	14.4	-89.5	14.5	3218.1
Lateral occipital cortex	L	younger	111	-13.3	-91.1	-17.0	2816.8
		older	131	-26.2	-91.6	4.8	3080.0
	R	younger	108	7.8	-98.0	5.0	1968.6
		older	292	13.5	-93.9	-14.7	3945.7
Lingual gyrus	L	younger	120	-4.2	-75.1	7.7	3727.8
		older	122	-0.1	-80.8	-5.4	3091.6
	R	younger	151	5.8	-73.4	10.6	3656.1
		older	183	14.8	-86.3	-11.1	4486.2
Pericalcarine	L	younger	99	-2.9	-75.7	8.0	3675.2
		older	91	-18.2	-76.3	7.5	3645.5
	R	younger	107	3.0	-73.3	10.9	4371.6
		older	114	15.4	-92.9	-11.7	3913.7
Fusiform gyrus	R	younger	30	23.3	-70.7	-10.7	1817.7
		older	75	28.4	-59.8	-18.0	3347.5
Updating							
300-400 ms							

Caudal anterior cingulate	L	younger	21	-3.9	13.3	36.6	1925.0
Posterior cingulate	L	younger	33	-0.5	5.9	40.0	1636.2
	R	younger	53	2.7	-35.4	36.9	1611.4
Paracentral lobule	R	younger	23	3.8	-24.6	56.4	1710.0
Superior frontal gyrus	L	younger	57	-6.6	21.6	45.2	2135.2
Cuneus	L	younger	30	1.2	-76.6	14.0	4516.0
		older	62	0.9	-85.2	27.0	2705.4
	R	younger	22	2.5	-77.5	17.2	3331.5
		older	73	3.1	-85.1	25.7	2551.4
Lateral occipital cortex	L	older	30	-5.5	-96.2	24.7	2434.6
	R	older	32	12.6	-100.0	15.9	1871.6
Lingual gyrus	L	older	22	-10.6	-47.6	-6.7	1410.8
	R	older	108	8.7	-75.8	-5.5	1896.5
Pericalcarine	L	younger	25	-2.9	-75.7	8.0	2724.6
	R	older	67	6.5	-80.8	4.4	1574.5
Precuneus	L	older	29	-2.3	-77.4	49.8	2076.8
	R	younger	36	9.4	-37.4	37.6	1679.3
		older	74	2.8	-72.1	47.4	2035.5
Superior parietal lobule	L	older	82	-0.4	-89.3	26.8	2557.4
	R	older	123	9.9	-74.7	57.1	2597.6
Updating							
400-1000 ms							
Superior frontal gyrus	L	younger	25	-5.5	60.5	34.9	2460.6
Cuneus	L	younger	53	1.2	-76.6	14.0	9828.2
		older	78	0.9	-85.2	27.0	1536.8
	R	younger	37	2.5	-77.5	17.2	7023.1
		older	51	3.1	-85.1	25.7	1453.2
Lateral occipital cortex	L	older	66	-11.3	-99.0	21.0	1726.2
Lingual gyrus	L	younger	70	-0.6	-72.7	9.3	5581.1
		older	70	-10.6	-47.6	-6.7	1039.6
	R	younger	78	5.8	-73.4	10.6	5417.9
		older	20	8.8	-78.3	-7.2	944.2
Pericalcarine	L	younger	57	-2.9	-75.7	8.0	5084.9
		older	79	-8.5	-84.8	6.4	1117.9
	R	younger	39	3.0	-73.3	10.9	7245.9
Superior parietal lobule	L	older	80	-10.3	-89.8	26.3	1722.9
	R	older	105	30.9	-55.8	52.0	1840.2
Fusiform gyrus	L	older	24	-20.8	-46.6	-17.4	1038.1
Substitution							
250-600 ms							
Isthmus cingulate	L	younger	50	-6.4	-41.7	23.2	945.0
		older	23	-0.1	-37.0	31.3	732.6
	R	younger	22	1.9	-42.4	23.6	764.7
		older	24	6.2	-31.9	26.3	784.6
Posterior cingulate	L	younger	54	-1.6	-9.2	29.7	786.7
	R	younger	47	2.1	-2.2	31.2	773.2
		older	26	7.2	-23.5	27.6	849.1
	L	younger	49	-0.5	20.5	62.0	884.1

Superior frontal gyrus	R	younger	91	10.4	24.6	66.0	1081.0
		older	20	3.2	33.2	58.2	755.7
Cuneus	R	older	23	6.3	-81.3	40.8	1137.9
Precuneus	L	younger	59	-8.9	-50.3	29.1	892.0
		older	144	-0.4	-77.3	40.1	1118.9
	R	older	101	5.5	-77.7	44.4	1196.5
Superior parietal lobule	L	younger	64	-11.2	-72.2	60.6	852.2
		older	86	-1.4	-83.2	37.9	1058.1
	R	older	162	6.2	-77.9	46.7	1189.6
Substitution							
550-750 ms							
Isthmus cingulate	L	younger	39	0.0	-46.6	31.4	868.4
	R	younger	39	2.2	-46.8	33.6	862.1
		older	23	7.9	-31.4	26.5	1084.3
Posterior cingulate	R	older	24	7.2	-23.5	27.6	1198.7
Caudal middle frontal gyrus	L	younger	24	-40.6	21.5	51.1	908.8
Cuneus	L	older	25	-0.7	-80.3	36.4	1584.4
	R	older	23	7.7	-79.2	40.9	1666.8
Inferior parietal lobule	R	younger	36	55.7	-62.0	20.7	1222.0
Precuneus	L	younger	85	-7.7	-53.0	27.7	858.2
		older	81	-0.4	-77.3	44.2	1725.0
	R	younger	61	3.9	-50.2	34.0	840.9
		older	98	5.5	-77.7	44.4	1814.1
Superior parietal lobule	L	older	71	-1.4	-83.2	37.9	1563.8
	R	younger	52	15.5	-88.3	39.9	916.6
		older	178	6.2	-77.9	46.7	1853.1
Fusiform gyrus	L	younger	21	-42.0	-29.3	-20.7	845.5
Gate opening							
150-250 ms							
Isthmus cingulate	L	younger	33	-0.3	-39.1	25.9	740.7
		older	53	-2.5	-55.6	6.1	1984.6
	R	younger	47	7.9	-31.4	26.5	802.8
		older	61	5.9	-51.8	4.9	1116.4
Posterior cingulate	R	younger	37	8.1	-28.5	27.4	810.1
		older	22	6.3	-5.0	30.9	738.4
Paracentral lobule	R	younger	28	11.9	-24.0	42.7	767.7
Lateral occipital cortex	L	younger	22	-2.2	-102.2	1.7	732.7
Lingual gyrus	L	older	65	-3.9	-57.9	2.3	1690.7
	R	older	122	3.7	-59.0	8.0	1522.2
Pericalcarine	R	older	38	8.8	-61.8	9.0	1140.0
Precuneus	L	younger	32	-6.2	-54.7	71.5	798.8
		older	78	-3.6	-57.9	6.1	1993.4
	R	younger	73	5.4	-50.3	70.4	853.9
		older	52	1.8	-58.5	15.0	1580.8
Superior parietal lobule	L	younger	35	-11.3	-55.8	72.8	804.0
	R	younger	50	18.8	-53.0	75.2	875.6

Fusiform gyrus	R	older	40	27.9	-61.8	-6.6	928.3
Gate opening							
300-500 ms							
Isthmus cingulate	L	younger	75	-0.3	-39.1	25.9	1654.0
	R	younger	54	1.8	-37.7	26.8	1674.7
		older	21	12.7	-56.1	6.5	819.9
Posterior cingulate	L	younger	36	-0.4	-28.8	27.6	1529.1
	R	younger	81	8.1	-28.5	27.4	1662.7
		older	23	4.4	-7.2	29.9	714.5
Paracentral lobule	R	younger	38	11.9	-24.0	42.7	1418.4
Cuneus	L	older	50	-0.4	-92.7	20.0	903.7
	R	older	76	6.7	-77.7	22.1	922.8
Lingual gyrus	R	older	94	3.7	-59.0	8.0	939.6
Pericalcarine	R	older	53	12.3	-72.9	15.2	935.4
Precuneus	L	younger	42	-3.7	-38.0	35.3	1413.6
		older	55	-0.1	-58.6	12.5	956.5
	R	younger	47	8.5	-36.3	37.5	1524.2
		older	57	4.4	-58.5	9.3	943.4
Superior parietal lobule	L	younger	20	-32.6	-35.7	50.1	1466.1
		older	25	-14.9	-87.1	26.2	973.2
	R	younger	26	36.7	-49.0	50.5	1573.0
		older	59	19.5	-89.3	36.8	880.4
Gate opening							
500-700 ms							
Isthmus cingulate	L	younger	71	-12.3	-52.9	3.8	1493.5
	R	older	40	12.7	-56.1	6.5	1007.9
Caudal middle frontal gyrus	R	older	20	41.1	19.6	53.7	815.9
Superior frontal gyrus	L	younger	21	-3.0	63.6	-1.0	1340.2
	R	younger	21	4.1	66.5	-3.6	1419.3
Cuneus	L	older	27	0.6	-71.8	19.7	1013.1
	R	older	38	8.7	-59.6	12.5	1025.8
Lingual gyrus	R	older	95	13.9	-63.5	5.2	1125.0
Pericalcarine	R	older	55	12.3	-72.9	15.2	1178.0
Precuneus	L	older	29	-0.1	-58.6	12.5	997.4
	R	older	46	4.4	-58.5	9.3	1072.0
Superior parietal lobule	L	older	22	-14.9	-87.1	26.2	1730.1
Gate closing							
150-300 ms							
Pars opercularis	L	younger	20	-48.1	29.6	17.7	1118.1
Pars triangularis	L	younger	31	-46.5	30.1	13.7	1143.5
Caudal middle frontal gyrus	L	younger	26	-38.1	0.2	33.6	1764.3
Rostral middle frontal gyrus	L	younger	443	-39.1	46.3	28.3	1346.3
Superior frontal gyrus	L	younger	85	-25.8	43.1	43.6	993.7
Cuneus	L	older	52	-1.8	-99.0	13.7	728.7
	R	older	53	14.4	-89.5	14.5	731.7
Lateral occipital cortex	L	older	114	-31.7	-95.1	7.6	746.9
	R	older	30	15.3	-92.5	15.4	758.6

Inferior parietal lobule	L	older	68	-41.0	-76.0	35.7	964.5
Superior parietal lobule	L	older	26	-12.8	-86.4	23.5	737.8
	R	older	31	15.8	-85.3	26.1	752.9
Inferior temporal gyrus	L	older	29	-47.3	-46.9	-27.9	933.8
Gate closing							
300-600 ms							
Posterior cingulate	L	younger	26	-9.4	-35.9	48.4	1041.7
		older	66	-0.1	-20.3	36.4	1186.8
	R	younger	22	1.5	-5.9	40.1	983.8
		older	80	2.1	-16.6	36.0	1154.2
Caudal middle frontal gyrus	R	older	28	41.2	0.9	38.8	994.0
Rostral middle frontal gyrus	L	younger	43	-27.3	49.7	36.6	1210.4
Paracentral lobule	R	older	45	5.5	-10.4	60.2	1169.0
Precentral gyrus	R	younger	21	21.3	-11.4	65.0	1057.7
		older	35	43.9	0.9	37.5	1007.4
Superior frontal gyrus	L	younger	73	-20.9	57.5	32.9	1218.4
		older	39	-0.2	-7.9	52.4	986.0
	R	younger	90	16.3	47.3	35.3	1232.6
		older	112	5.3	-9.8	61.2	1181.2
Lateral occipital cortex	L	older	34	-34.8	-77.8	-11.4	1381.7
Superior parietal lobule	R	older	24	15.8	-85.3	26.1	1001.8
Inferior temporal gyrus	L	older	24	-47.3	-46.9	-27.9	1545.3
Fusiform gyrus	L	older	24	-46.0	-45.1	-29.0	1561.2
